# The Goldilocks Paradox of Bioelectronics: Misreporting Piezoresistive Gauge Factor Is Obstructing Research Advancements

**DOI:** 10.1002/adma.202503746

**Published:** 2025-06-12

**Authors:** Conor S. Boland

**Affiliations:** ^1^ School of Mechanical and Manufacturing Engineering Dublin City University Dublin 9 Glasnevin Ireland; ^2^ RAPID Institute Dublin City University Dublin 9 Glasnevin Ireland

**Keywords:** bioelectronics, gauge factor, nanocomposites, standards for reporting, strain sensor

## Abstract

In the fable Goldilocks and The Three Bears, Goldilocks discerns between seemingly similar bowls of porridge using temperature. In the field of wearable bioelectronics, researchers do not have the same luxury of simple comparative analysis. Devices based on composite materials applying electrically conductive nanomaterial and polymer networks present a technology path towards an Internet of Things driven healthcare system. However, research progress has been obstructed by a lack of consistency with regards to the industry standard performance metric, the gauge factor (*G*). Paradoxically, studies cannot be compared, as no one study measures *G* the same. However, extrapolating the correct value for *G*, its intrinsic relationship between the signal linearity figure of merit outlines fieldwide performance limitations for devices, new metrics and materials selection criteria.

## Graphical abstract

The misreporting of piezoresistive gauge factor for bioelectronic devices impacts the ability of researchers to assess the status of the field. Like Goldilocks, researchers need the ability to take similar bowls of porridge, or devices, and perform “taste” tests. A systematic analysis of published data outlines reporting and performance standards that must be adhered to by editors and researchers alike.

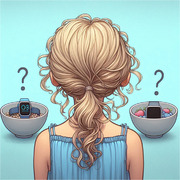

## 1 Introduction

Overcoming commercial boundaries has been a challenge for all aspects of nanotechnology. However, strides have been made to counteract this issue through the demonstration of standardized reporting across several key research areas, such as material characterization,^[^ ^1^ ^]^ energy storage,^[^ ^2^ ^]^ clean energy harvesting,^[^ ^3^ ^]^ nano–bio^[^ ^4^ ^]^ and mechanical reinforcement.^[^ ^5^ ^]^ One field that would greatly benefit from a similar treatment is the study of polymer‐based nanocomposites applied as wearable bioelectronics. For these devices, they have been demonstrated as effectively measuring touch, joint movement, muscular flexation, respiration, heart function, wound monitoring, and skin temperature all as a function of time.^[^ ^6^ ^]^

What is most peculiar about the research area is that if one takes for example the three most cited papers on nanocomposite bioelectronics in 2024 according to Scopus.^[^ ^7^ ^]^ Like Goldilocks and The Three Bears, our three bowls of porridge (or studies) in appearance are very similar (i.e., all perform similar measurements). So, by what means can Goldilocks (the potentially nonexpert reader) discern between the different porridge? Well, a figure of merit is required. Where Goldilocks in the story used temperature, readers can use the industry standard sensitivity metric, the gauge factor (*G*). However, herein lies the paradox: all three above named studies report *G* differently. It begs the question: which study reported *G* correctly? Surprisingly, the answer is that none of them have. In essence readers are denied the ability to perform basic comparative analysis on such studies, as a standard for reporting is lacking. Most damaging is that this lack of standardized reporting has a cascade effect, as it sets a bad precedent in which one would go about analyzing their work. As such, the misreporting of *G* goes far beyond these three examples and is an issue that has become fieldwide. The culmination of this misreporting is the dissemination of wildly inaccurate values that have no research value, which have distorted editorial and the reader's view of the state‐of‐the‐art.

Most challenging though, is the limitations misreporting places upon researcher's ability to develop models that facilitate an understanding of the electromechanical mechanisms in nanocomposites, which control performance. Such models would broadly be invaluable tools in developing modes in which properties can be optimized to better fit their application. However, if a model is developed based on a unique descriptor of *G*, rather than a standard, it cannot be widely applicable to other studies. The reason being that the description of *G* in such studies is unique to that one system. Conversely, attempting to develop a model based on the industry standard descriptor also is relatively difficult in the research field's current state, due to most studies reporting *G* differently. Thus, it would require a systematic review of works to extrapolate true values individually. Goldilocks and the Three Bears emphasizes Goldilocks's desire to find balance or options that are “just right” to suit her needs. Similarly, researchers need to seek a balance between objective analytical descriptions of devices and the requirements for device applications. The moral lesson of the fable is that consequences of actions need to be highlighted. Here, the goal of the perspective is comparable to the bears, not to scare Goldilocks (i.e., researchers), but to bring light to the fact that current reporting standards are lacking and this ongoing trend is obstructing research. Through a systematic review of recent published works here, >80% of studies was found to misreport upon an overestimated value for *G*.

## 2 Background on Gauge Factor

Historically, *G* is a figure of merit dating back to studies on the piezoresistive properties of metals in the early 1900s.^[^ ^8^ ^]^ In present day, *G* is still used as an industry standard and is applied to quantify the performance of commercial sensors. These devices work on the principle of a metal foil under applied strain (*ɛ*) deforming, resulting in a linear change in device electrical resistance (Δ*R*).^[^ ^8^, ^9^ ^]^ The Δ*R* can then be converted to strain using what is known in industry as the K‐factor or gauge factor (sometimes written as gage factor).^[^ ^10^ ^]^ To extrapolate *G*, a simple linear expression can be used^[^ ^8^, ^11^ ^]^

(1) $$G=\frac{\Delta R}{R_{0}}\frac{1}{\varepsilon }$$

where *R*_0_ is zero‐strain resistance. Essentially, a linear fit that passes through the origin is applied to Δ*R*/*R*_0_ versus *ɛ* data beginning at low strain. The slope of this line fit would then be the value for *G*. Fundamentally, values for *G* must be very finely reported in industry, so as an accurate product is distributed to consumers. With the wrong metric value affecting the reliability of a device.^[^ ^12^ ^]^ Thus, research wanting to reach the commercial arena must observe this standard for reporting.

Due to their mechanical compliance, commercial devices are too rigid to comfortably conform to the body.^[^ ^13^ ^]^ Thus, the solution to realizing wearable sensing electronics is soft, highly elastic strain sensing nanocomposites that make use of flexible polymers.^[^ ^6^ ^]^ Similar to metal foil sensors, nanocomposite electrical resistance responds to applied strain linearly (beginning at small strain values). This allows *G* to also be used to transduce electrical signal to strain, while also enabling direct performance comparisons with commercial devices. The advantage of these nanocomposites beyond their compliable nature are the strain‐induced changes in resistivity (Δ*ρ*), resulting in very large *G* values. More intuitively, Equation (1) can be written as^[^ ^8^, ^11^ ^]^

(2) $$G=1+2\nu +\frac{\Delta \rho }{\rho_{0}}\frac{1}{\varepsilon }$$

where *ν* is Poisson's ratio and *ρ*_0_ is zero‐strain resistivity. Essentially, *G* can be described as having a dependence on both the dimensions and resistivity of a material. For commercial sensors, their response is primarily reliant on dimensional changes (i.e., Δ*ρ*/*ρ*_0_ ≈ 0). For these devices, *ν* ≈ 0.5, resulting in *G* ≈ 2 being a commercial benchmark.^[^ ^9^ ^]^ However, on average, nanocomposite devices present values of *G* ≈ 40 due to large Δ*ρ*/*ρ*_0_ values.^[^ ^14^ ^]^ Electrical current flow in these devices is reliant on percolative‐like networks of conductive materials. As nanocomposites undergo deformation, they will have a linear Δ*R*/*R*_0_ occur until a critical strain limit, known as the working factor (*W*).^[^ ^14^, ^15^ ^]^ Essentially, *W* is the strain limit for the fitting of *G*, where data begin to deviate from linearity. Fundamentally, *W* describes the elastic working range of a sensor in which measurements can be converted from electrical to strain outputs using a device's respective *G* value. This differs from the maximum strain at which a nanocomposite ceases to conduct current, which occurs in the plastic regime of the devices mechanical properties. In this region, response can no longer be described by *G*. Mechanistically, this critical strain limit signifies a crossover in the resistive mechanism from conductor areal overlap changing (linear Δ*R*/*R*_0_) to conductor–conductor tunneling (exponential Δ*R*/*R*_0_) due to plastic deformation.^[^ ^16^ ^]^ The magnitude of *W* is related to the yield strain of a nanocomposite, with said value tuneable via the density of the nanonetwork^[^ ^17^ ^]^ and/or the conductivity of the polymer matrix.^[^ ^18^ ^]^ Essentially, increasing the density of the network in the confined volume of nanocomposite results in the number of overlapping constituents to increase due to confinement.^[^ ^16^ ^]^ Similarly, increasing the conductivity of a matrix essentially shorts the nanonetwork circuit, decreasing the potential for charge carriers to tunnel between constituents.^[^ ^14a^ ^]^ To note, increasing density and matrix conductivity are widely reported to have a negative correlation with the resultant value of *G*.

## 3 Misreporting in Literature

With respect to current research and its misreporting of *G*, these fittings generally involve the following issues.

Issue #1: Gauge factor changing with every strain data point

**Figure 1:**
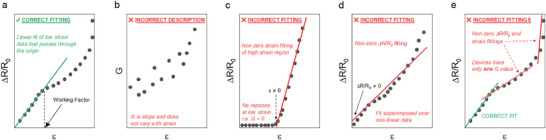
Ways in which data are fitted correctly and incorrectly. Visualization of a) the correct and b–e) the incorrect ways in which gauge factor (*G*) is extrapolated or presented from fractional resistance change (Δ*R*/*R*_0_) versus strain (*ɛ*) plots. Plots depicting the incorrect fittings show issues in order of #1 to #4.

The metric *G* describes the slope of the initial linear regime of a Δ*R*/*R*_0_ versus *ɛ* plot (**Figure** **1**a). Essentially, the data is a straight line, and the slope of a given line does not change unless the data changes. However, in some reports, *G* was shown to have a value that changes with strain.^[^ ^19^ ^]^ This appears to stem from the misuse of Equation (1) to convert every data point or groupings of data points in Δ*R*/*R*_0_ versus *ɛ* plots into a *G* value (Figure 1b). Essentially, the issue here is the misrepresentation of what *G* actually is and what it means. Fundamentally, a system has only one *G* value.

Issue #2: Nonzero strain fitting

For Equation (1), it is a linear expression with an intercept of zero. However, many papers report values for *G* that are extrapolated with fits that do not pass through the origin (Figure 1c).^[^ ^19^, ^20^ ^]^ Essentially, the equation used to calculate *G* in these studies takes the form of

(3) $$S=\Delta R/R_{0}\cdot 1/\varepsilon -C/\varepsilon$$

where *C* is a positive, nonzero intercept, and *S* is the slope of the line. According to Equation (1) and industry, this *S* value is not *G*. In some cases, the devices even appear to have no electromechanical response at low strain,^[^ ^19^, ^21^ ^]^ with increases in Δ*R*/*R*_0_ at high strains appearing to be attributed to a failure mechanism in the device. In these aforementioned studies, Equation (3) was applied. However, it certainly is appropriate for studies to quote a rate of change in Δ*R*/*R*_0_ with respect to *ɛ* when examining the mechanical compliance of their materials or device. However, it is not appropriate to call this quantity *G*.

Issue #3: Superimposed fitting

This issue involves data in which there are clear linear and exponential regimes in the data that are ignored. Through this method, a linear fit is placed across the entirety of the Δ*R*/*R*_0_ versus *ɛ* data plot (Figure 1d).^[^ ^19^, ^22^ ^]^ Due to either low data sampling^[^ ^20^, ^22^, ^23^ ^]^ and/or the wide strain range^[^ ^24^ ^]^ that was investigated, *R* ^2^ values approaching 1 are reported. Though in actuality, this is not the case and the value for *G* reported is incorrectly extrapolated. Many of these superimposed fits also incorporate aspects of Issue #2, where nonzero *x*‐ and *y*‐intercepts are noted.

Issue #4: Multifitting of data curves

This phenomenon is a culmination of the three previous issues. Essentially this involves fitting the low strain regime of the Δ*R*/*R*_0_ versus *ɛ* either correctly or using the Issue #2 or #3 methods. Again, using Issue #2 and #3 methods, exponential regions of the data have linear fits applied (Figure 1e). As per the description, this essentially results in authors audaciously quoting two,^[^ ^19^, ^20^, ^22^, ^23^, ^25^ ^]^ three,^[^ ^19^, ^20^, ^22^, ^23^, ^24^, ^26^ ^]^ four,^[^ ^20^, ^22^, ^27^ ^]^ five,^[^ ^28^ ^]^ six,^[^ ^29^ ^]^ seven,^[^ ^19n,o^ ^]^ and even eight^[^ ^30^ ^]^ values for *G*. Each one of these values is then designated to a zone or strain region in which they describe the response curve. Much of the problem with this method is the low level of data sampling that occurs, with these “regions” having the appearance of being linear. Whereas, with proper data sampling, this would not be the case. Simply put, a device can only have one *G*. Most damaging about this method is the quoting of the high strain fittings, where devices are essentially approaching failure. In these regions, values for *G* are unrealistically large and skew the reader's view of the research landscape. When these high strain values are compared to the true *G* values, there are often several orders of magnitude difference between the two.

Most egregious is many of these same studies that apply the above‐mentioned methodologies then use the commercial benchmark of *G* ≈ 2 to convey perspective with regards to their misreported metric values. These miscalculated values in literature are then reported alongside a maximum strain value, which corresponds to the limit at which the device no longer is electrically conductive. These methods of performance description lead to the creation of literary review plots with no discernible trends or research value (i.e., data clouds).^[^ ^19^, ^31^ ^]^

## 4 Systematic Analysis of Reported Data

**Figure 2:**
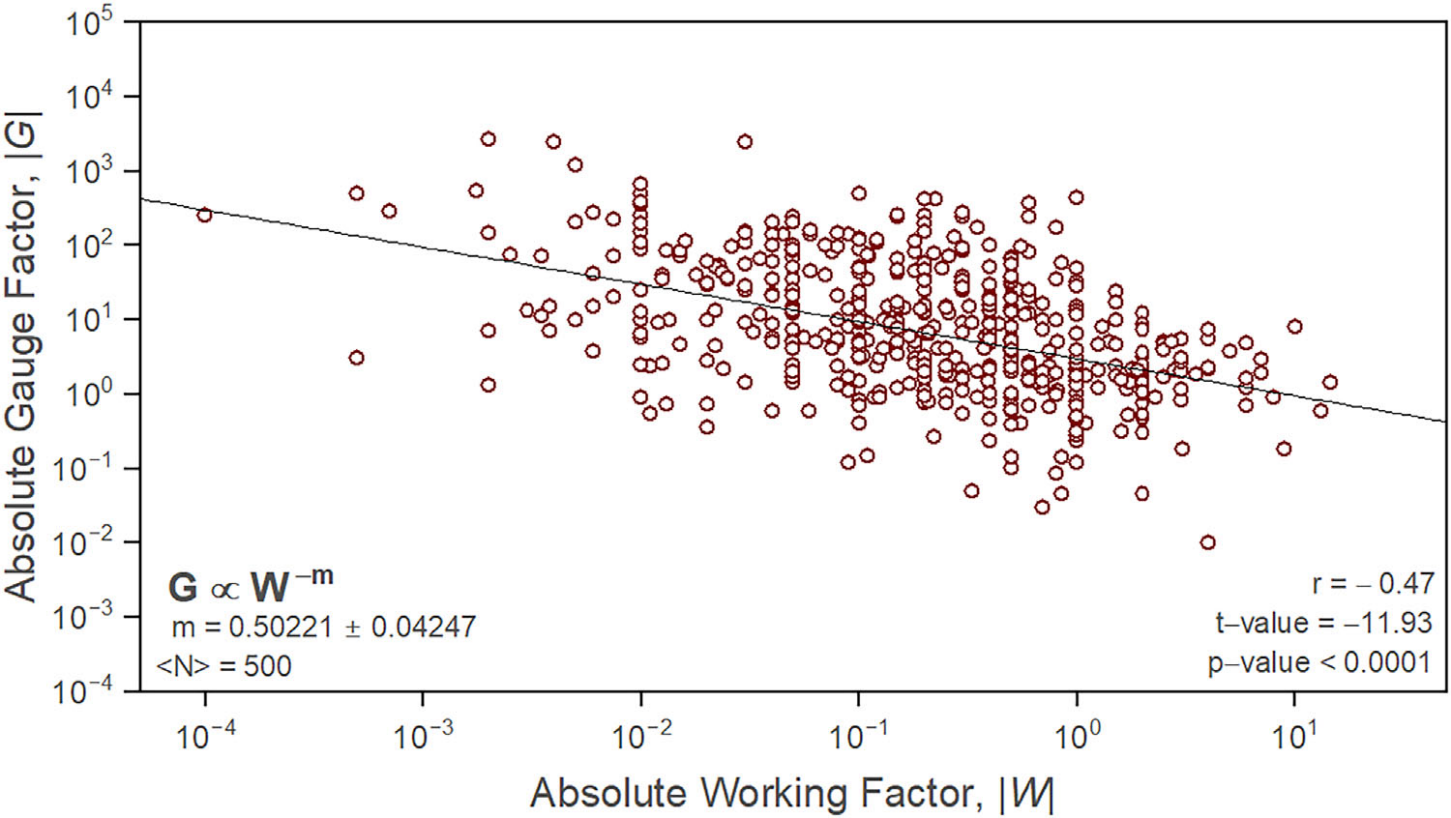
Literary data master plot. A plot of the absolute value of gauge factor (|*G*|) as a function of absolute working factor value (|*W*|) extrapolated from literary data in Table S1 and refs. 13 and 14 of the Supporting Information, totaling 500 data points. Data were found to scale according to a power law with an exponent of −0.50221 ± 0.04247, with the fit having an *r*‐value of −0.47, a *t*‐value of −11.93 and a *p*‐value <0.0001; corroborating a statistically correlated and significant model.

When sampling 239 recent studies which reported upon *G* and a nanocomposite device for bioelectronic applications, astonishingly only ≈19.6% correctly reported the metric. Albeit many that did report correctly were found to do so with suboptimal data sampling (Table S1, Supporting Information). The absolute percentage difference between the true values for *G* and those reported by the studies differed by 56.81% ± 37.83%. This is in addition to the 234.70% ± 95.52% difference in true and reported functional working strain values for devices (Figure S1, Supporting Information). However, when the true values for *G* are plotted against their respective *W* value in **Figure** **2**, a well‐defined power‐law with an exponent of −0.50221 ± 0.04247 was observed across a dataset containing 500 individual studies. Here, the exponent is related to Kraus's universal constant (*m*), which has a value of 0.5 in composite materials.^[^ ^16^, ^32^ ^]^ For the presented fit, values for the Pearson correlation coefficient (*r*), the test statistic (*t*‐value), and probability value (*p*‐value) were −0.47, −11.93, and <0.0001, respectively. These values indicate a statistically correlated and significant model. For the research landscape of nanocomposite bioelectronics, the relationship between *G* and *W* presents foundations in which systems can be engineered to better meet specific application requirements and commercial expectations. Fundamentally, the presented scaling sets device limitations, whereby large values for *G* will always result in a smaller linear sensing range and vice versa. This limitation stems from *W*’s direct relationship with the intrinsic yield strain of a material through the Kraus model via *W* ≈ *C* ^1/2^ *^m^*,^[^ ^33^ ^]^ where *C* is a constant related to the ratio between network bonds reforming and breaking in a nanonetwork during deformation. Intuitively, *C* is inversely proportional to *G*, as electromechanical sensitivity is controlled by the rate at which network connections break as a function of strain. Essentially, rapid bond breaking will yield a small *C* value. Due to the noted proportionalities, such a scenario would result in a large *G* and subsequently small *W* values. Similarly, large *C* values result in small *G* and large *W* values. The presentation of this significant relationship between the two metrics sets precedence for not only the need but also the requirement for standardized reporting with regards to bioelectronic devices in research.

## 5 A Call for Publishing Standards

With regards to actionable steps to facilitate compliance with the call for standardized reporting discussed here, measurement and displayed figure requirements must be put in place. With regards to testing the electromechanical response of a material or device, a minimum sampling of one data point for every 1% strain applied must be adhered to. Fundamentally, more data points facilitate more accurate fittings of response curves. For the electromechanical properties, ∆*R*/*R*0 versus *ɛ* data plots must be shown. With special attention given to the low‐strain regime, so as linear trends are clearly presented. Such plots should be required for all material and device variations that are presented, discussed or applied in a study. All plots must then be fitted, beginning at low strain and the fit line passing through the origin, with Equation (1) to facilitate an extrapolation and quoting of *G* and *W* values. From these fits, *G* would be the slope of the line and *W* the strain limit at which the data begins to deviate from the linear fitting. The reporting of both values is of the upmost importance, as *W* reflects the strain value a device can measure up to. While *G* is the description of how accurately this strain range can be measured. These requirements naturally need to be enforced by journals and editors through revised author guidelines, data reporting check lists, and/or additional data requirements sections in manuscripts.

### 5.1 Statistical Analysis

For the statistical methodology, the program Origin Labs 2024 was applied to process and plot data.

## Conflict of Interest

The authors declare no conflict of interest.

## Supporting information



Supporting Information

## Data Availability

All data are available in the main text or in the Supporting Information.
