# Supplementary material for: The Goldilocks Paradox of Bioelectronics: Misreporting Piezoresistive Gauge Factor Is Obstructing Research Advancements
Source: Adv Mater. 2025 Jun 12;37(34):2503746. doi: 10.1002/adma.202503746 (PMC12392876; doi:10.1002/adma.202503746)
Supplement: Supplementary file 1 — Supporting Information [file ADMA-37-2503746-s001.docx]

**Supplementary Materials**

**The Goldilocks Paradox of Bioelectronics – Misreporting Piezoresistive Gauge Factor is Obstructing Research Advancements**

Conor S. Boland

Corresponding author: [conor.boland@dcu.ie](mailto:conor.boland@dcu.ie)

File includes:

Figure S1

Table S1

Notes

References (1–239)

**Figure**

**
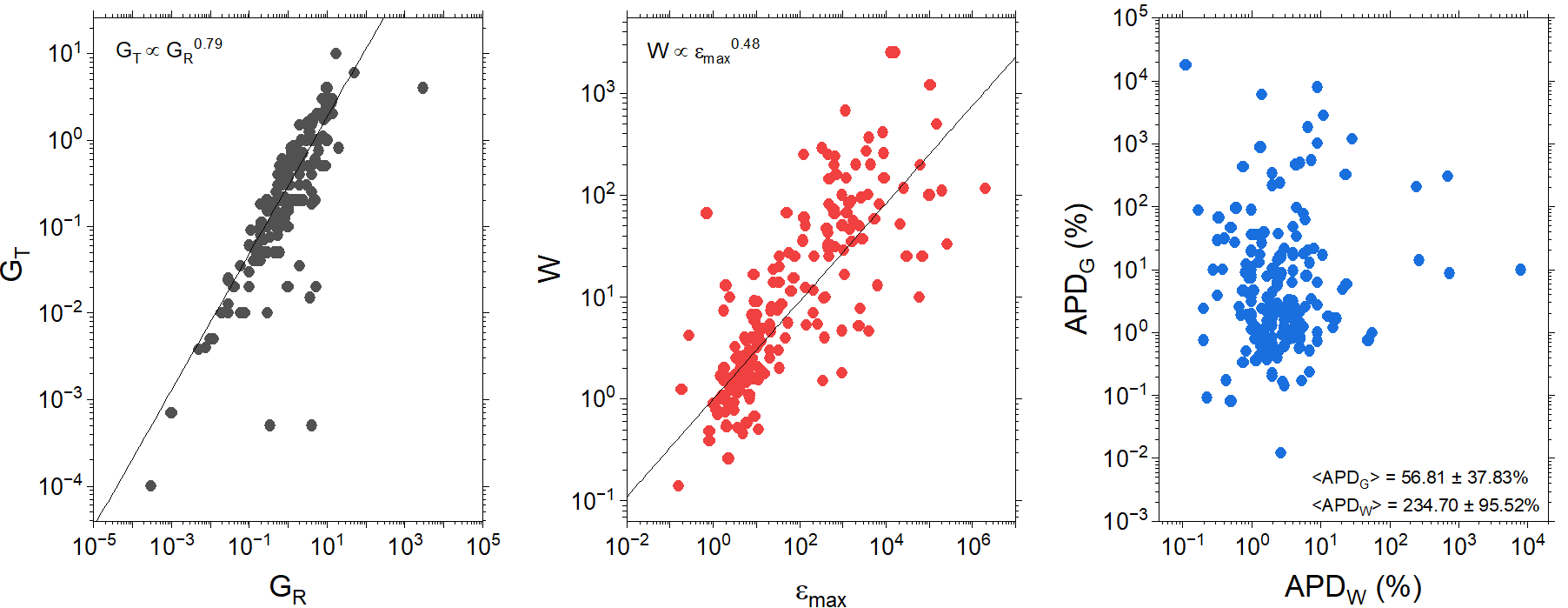
Figure S1: Literary data from select publications.** (**a** and **b**) Absolute true gauge factor (*G*_true_) and absolute the critical strain limit of the linear gauge factor fit (*i.e.* working factor, *W*) plotted against absolute reported gauge factor (*G*_reported_) and absolute maximum strain (ε_max_) respectively. (**c**) The absolute percentage difference (APD) between *G*_true_ and *G*_reported_ (*APD*_G_) as a function of *W* and ε_max_ (*APD*_W_). Average values of the quantities are <*APD*_G_> = 56.81 ± 37.83% and <*APD*_W_> = 234.70 ± 95.52%.

**Table**

| Reference | Reported \|ε_max_\| | Reported \|*G*_R_*\|* | \|*W\|* value | True \|*G*_T_*\|* | Issue |
| --- | --- | --- | --- | --- | --- |
| ^[1a]^ | 0.3 | 2 | 0.05 | 13 | #1 |
| ^[1b]^ | 0.25 | 477 | 0.05 | 25 | #3 |
| ^[1c]^ | 4 | 32.5 | 0.0005 | 3 | #1 |
| ^[1d]^ | 0.2 | 1006 | 0.1 | 50 | #1 and #2 |
| ^[1e]^ | 0.5 | 1.00E+05 | 0.05 | 100 | #1, #2, and #4 |
| ^[1f]^ | 0.3 | 11 | 0.1 | 9 | #1 |
| ^[1g]^ | 0.6 | 70000 | 0.05 | 25 | #1, #2 and #3 |
| ^[1h]^ | 0.7 | 2.3 | 0.3 | 1.6 | #1 and #3 |
| ^[1i]^ | 0.5 | 6.7 | 0.25 | 2.4 | #1 |
| ^[1j]^ | 0.0025 | 18300 | N/A | N/A | #1 and #2 |
| ^[1k]^ | 4 | 7.3 | 0.4 | 1 | #1 and #2 |
| ^[1l]^ | 1 | 1251 | 0.15 | 66.67 | #1 |
| ^[1m]^ | 0.55 | 10 | 0.3 | 6.67 | #1 and #3 |
| ^[1n]^ | 10 | 0.83 | 1 | 0.48 | #1 and #4 |
| ^[1o]^ | 0.56 | 9178.62 | 0.08 | 146 | #1 and #4 |
| ^[1p]^ | 0.7 | 25 | 0.4 | 18.75 | #1 |
| ^[1q]^ | 1.78 | 5.98 | 0.75 | 3.73 | #1 |
| ^[1r]^ | 7.5 | 1903 | N/A | N/A | #1 and #2 |
| ^[1s]^ | 1.6 | 217 | 0.6 | 25 | #1 |
| ^[1t]^ | 1.3632 | 5536.346 | 0.8536 | 57.894 | #1 and #4 |
| ^[1u]^ | 9 | 31.51 | 2 | 7.4 | #1 |
| ^[1v]^ | 0.075 | 200000 | 0.01 | 110 | #1 |
| ^[1w]^ | 0.5 | 8.73 | 0.15 | 16.67 | #1 and #3 |
| ^[2a]^ | 3.7 | 971.7 | 0.015 | 4.66 | #2 and #4 |
| ^[2b]^ | 0.007623 | 12787 | 0.004 | 2500 | #2 |
| ^[2c]^ | 2 | 3.75 | 0.3 | 1.67 | #2 |
| ^[2d]^ | 0.15 | 673 | 0.05 | 240 | #2 |
| ^[2e]^ | 0.061 | 64 | 0.035 | 11.43 | #2 |
| ^[2f]^ | 0.2 | 14.5 | 0.1 | 4.9 | #3 |
| ^[2g]^ | 4 | 2.95 | 1.75 | 1.43 | #2 |
| ^[2h]^ | 0.68 | 468 | 0.3 | 33.33 | #2 and #4 |
| ^[2i]^ | 1.08 | 1443 | 0.3 | 83.3 | #2 |
| ^[2j]^ | 4.6 | 101.75 | N/A | N/A | #2 and #3, See note #4 |
| ^[2k]^ | 2.5 | 10.957 | 0.5 | 4 | #2 and #4 |
| ^[2l]^ | 0.55 | 33.99 | 0.09 | 13.88 | #2 and #3 |
| ^[2m]^ | 0.03 | 462 | 0.0235 | 42.55 | #2 and #4 |
| ^[2n]^ | 7.5 | 5.51 | 3 | 2.63 | #2 and #4 |
| ^[2o]^ | 1 | 9.21 | 0.17 | 5.88 | #2 and #4 |
| ^[2p]^ | 6 | 9.2 | 0.75 | 0.67 | #2 and #4 |
| ^[2q]^ | 4 | 11.2 | 1 | 0.5 | #2 |
| ^[2r]^ | 1 | 35 | 0.2 | 2 | #2 |
| ^[3a]^ | 0.008 | 200 | N/A | N/A | #2 |
| ^[3b]^ | 0.3 | 25.9 | N/A | N/A | #2 |
| ^[3c]^ | 4 | 2.1 | 0.25 | 0.96 | #4 |
| ^[3d]^ | 0.01 | 108241.7 | 0.005 | 1202.6 | #4 |
| ^[3e]^ | 0.02 | 20.92 | 0.01 | 2.5 | #3 |
| ^[3f]^ | 0.06 | 3400 | N/A | N/A | #2 |
| ^[3g]^ | 0.7 | 35 | N/A | N/A | #2 |
| ^[3h]^ | 1.1 | 902 | N/A | N/A | #2 |
| ^[4k]^ | 0.0003 | 126.7 | 0.0001 | 250 | #3 |
| ^[4a]^ | 0.6 | 2406 | 0.11 | 5.2 | #3 and #4, see note #1 |
| ^[4b]^ | 1.91 | 5.7 | 0.66 | 1.45 | #3 and #4 |
| ^[4c]^ | 0.001 | 338.9 | 0.0007 | 289 | #3 |
| ^[4d]^ | 1.5 | 140.4 | 0.5 | 12.4 | #3 and #4 |
| ^[4e]^ | 0.4 | 8.98 | 0.11 | 9.09 | #3 |
| ^[4f]^ | 0.2 | 140 | 0.05 | 50 | #3 and #4 |
| ^[4g]^ | 1 | 35 | 0.3 | 25 | #3 |
| ^[4h]^ | 0.11 | 1.515 | 0.09 | 1.67 | #3 and #4 |
| ^[4i]^ | 0.16 | 721.67 | 0.06 | 158.48 | #3 |
| ^[4j]^ | 0.2 | 4.3 | 0.05 | 2 | #3 |
| ^[5a]^ | 0.5 | 41 | N/A | N/A | #3, see note 3 |
| ^[5b]^ | 0.35 | 8 | 0.075 | 4 | #3 |
| ^[5c]^ | 8.34 | 3.74 | 1.72 | 0.52 | #3 and #4 |
| ^[5d]^ | 1.20 | 470 | 0.2 | 250 | #3 |
| ^[5e]^ | 17 | 22.95 | 10 | 7.95 | #3 and #4, see note #4 |
| ^[5f]^ | 1 | 5.47 | 0.375 | 4 | #3 and #4 |
| ^[6a]^ | 3 | 1.8 | 1 | 1.5 | #3 |
| ^[6b]^ | 0.8 | 7.747 | 0.21 | 6.66 | #3 |
| ^[6c]^ | 10 | 4.41 | 4 | 2.29 | #3 and #4, see note #4 |
| ^[6d]^ | 0.3 | 2.5 | 0.1 | 1.5 | #3 |
| ^[6e]^ | 1 | 2.46 | 0.02 | 10 | #3 |
| ^[6f]^ | 1 | 3.22 | 0.1 | 3.22 | #3 |
| ^[6g]^ | 4 | 1.51 | 0.5 | 1 | #3 and #4 |
| ^[7a]^ | 0.03 | 117.9 | 0.0125 | 34.8 | #4 |
| ^[7b]^ | 2.5 | 20.57 | 0.5 | 5 | #4 |
| ^[7c]^ | 0.3 | 1.84 | 0.2 | 2 | #4 |
| ^[7d]^ | 0.5 | 1.74 | 0.125 | 1.07 | #4 |
| ^[7e]^ | 1.5 | 2.74 | 0.7 | 1.22 | #4 |
| ^[7f]^ | 1 | 1.2 | 0.4 | 0.79 | #4 |
| ^[7g]^ | 0.1 | 129.3 | 0.02 | 60.1 | #4 |
| ^[7h]^ | 1.3 | 3.4 | 0.6 | 2.5 | #4 |
| ^[7i]^ | 0.2 | 400 | 0.08 | 10 | #4 |
| ^[7j]^ | 0.6 | 34.3 | 0.5 | 19.6 | #4 |
| ^[7k]^ | 4.5 | 3.623 | 1.7 | 1.152 | #4 |
| ^[7l]^ | 0.5 | 3.12 | 0.25 | 0.77 | #4 |
| ^[7m]^ | 0.45 | 4468.9 | 0.05 | 200 | #4 |
| ^[7n]^ | 10 | 8.82 | 2.5 | 3.91 | #4 |
| ^[7o]^ | 5.4 | 454 | 0.02 | 30 | #4 |
| ^[7p]^ | 0.55 | 3.08 | 0.2 | 0.93 | #4, see note #4 |
| ^[7q]^ | 11 | 2.67 | 3 | 0.84 | #4, see note #4 |
| ^[7r]^ | 8 | 2.4 | 2 | 1.5 | #4 |
| ^[7s]^ | 0.3 | 430.6 | 0.15 | 46.58 | #4 |
| ^[7t]^ | 0.55 | 3528 | 0.3 | 270 | #4 |
| ^[7u]^ | 4 | 480 | 0.18 | 82 | #4 |
| ^[7v]^ | 5.5 | 8.1 | 2 | 2.5 | #4 |
| ^[7w]^ | 4.5 | 1.26 | 1 | 0.7 | #4 |
| ^[7x]^ | 6 | 4.08 | 1 | 2.59 | #4, see note #4 |
| ^[7y]^ | 1 | 47.60 | 0.5 | 3.94 | #4 |
| ^[7z]^ | 0.4 | 24.7 | 0.19 | 13.9 | #4 and see note #4 |
| ^[7aa]^ | 0.55 | 5.07 | 0.4 | 0.46 | #4, see note #4 |
| ^[7ab]^ | 0.8 | 39 | 0.4 | 8.5 | #4, see note #4 |
| ^[7ac]^ | 0.005 | 207.95 | 0.0038 | 6.96 | #4 |
| ^[7ac]^ | 0.005 | 73.8 | 0.0038 | 15.31 | #4 |
| ^[7ad]^ | 1 | 0.83 | 0.5 | 0.39 | #4, see note #4 |
| ^[7ae]^ | 10 | 1.8 | 4 | 7.3 | #4 |
| ^[7af]^ | 0.49 | 0.28 | 0.13 | 4.17 | #4, see note #4 |
| ^[7ag]^ | 5 | 2.51 | 1 | 1.28 | #4 |
| ^[7ah]^ | 0.2 | 2.02E+6 | 0.18 | 115 | #4 |
| ^[7ai]^ | 0.03 | 121 | 0.025 | 36 | #4 |
| ^[7aj]^ | 0.65 | 58 | 0.3 | 27 | #4 and see note #4 |
| ^[7ak]^ | 13.04 | 10.96 | 2.7 | 5.14 | #4 |
| ^[7al]^ | 0.28 | 1493 | 0.2 | 46 | #4 |
| ^[7am]^ | 3.3 | 8.86 | 1.6 | 1.58 | #4 |
| ^[7an]^ | 0.3 | 30000 | 0.01 | 25 | #4 |
| ^[8a]^ | 0.3 | 25542 | 0.1 | 117 | #4 |
| ^[8b]^ | 1.1 | 519 | 0.3 | 32 | #4 |
| ^[8c]^ | 2.5 | 3.22 | 0.2 | 3.22 | #4 |
| ^[8d]^ | 1.34 | 210.55 | 0.58 | 11.59 | #4 |
| ^[8e]^ | N/A | N/A | 0.04 | 5 | #1 |
| ^[8f]^ | 0.02 | 2.227 | 0.01 | 0.884 | #4 |
| ^[8g]^ | 0.45 | 0.187 | 0.15 | 1.23 | #4 |
| ^[8h]^ | 4 | 4.39 | 1.5 | 1.74 | #4 |
| ^[8i]^ | 10 | 1.96 | 2 | 0.75 | #4 |
| ^[8j]^ | 0.21 | 616 | 0.11 | 73 | #4 |
| ^[8k]^ | 2 | 0.16 | 0.5 | 0.14 | #4 |
| ^[8l]^ | 10 | 3.17 | 2 | 1.74 | #4 |
| ^[8m]^ | 0.7 | 2.2E+4 | 0.4 | 51.4 | #4 |
| ^[8n]^ | 0.13 | 2000 | 0.04 | 200 | #4 |
| ^[8o]^ | 0.55 | 387 | 0.1 | 4 | #4 |
| ^[8p]^ | 0.15 | 960 | 0.04 | 100 | #4 |
| ^[8q]^ | 0.58 | 1591.2 | 0.05 | 87.84 | #4 |
| ^[8r]^ | 3000 | 54.5 | 4 | 5.6 | #4 |
| ^[8s]^ | 0.7 | 7178 | 0.6 | 82.16 | #4 |
| ^[8t]^ | 0.012 | 60000 | 0.005 | 9.98 | #4 |
| ^[8u]^ | 7 | 6.42 | 0.5 | 2.31 | #4 |
| ^[8v]^ | 2.5 | 354 | 0.7 | 1.5 | #4 |
| ^[8w]^ | 1 | 460.9 | 0.3 | 30.9 | #4 |
| ^[8x]^ | 1 | 7.03 | 0.3 | 1.09 | #4 |
| ^[8y]^ | 0.86 | 2632 | 0.55 | 94 | #4 |
| ^[8z]^ | 50 | 21.5 | 6 | 4.9 | #4 |
| ^[8aa]^ | 2 | 3.26 | 0.2 | 1.63 | #4 |
| ^[8ab]^ | 0.04 | 663.98 | 0.02 | 31.09 | #4 |
| ^[8ac]^ | 3.5 | 21.25 | 1.25 | 4.54 | #4 |
| ^[8ad]^ | 3 | 2.06 | 0.3 | 0.55 | #4 |
| ^[8ae]^ | 0.8 | 2.3 | 0.22 | 0.26 | #4 |
| ^[8af]^ | 0.8 | 1.04 | 0.125 | 0.888 | #4 |
| ^[8ag]^ | 10 | 21.12 | 3 | 5.42 | #4 |
| ^[8ah]^ | 8 | 11.17 | 0.5 | 4.21 | #4 |
| ^[8ai]^ | 1.2 | 146 | 0.5 | 5.3 | #4, see note #4 |
| ^[8aj]^ | 9 | 10.42 | 0.5 | 3.92 | #4 |
| ^[8ak]^ | 0.1 | 16021 | 0.03 | 2500 | #4 |
| ^[8al]^ | 14 | 3.76 | 3 | 1.14 | #4 |
| ^[8am]^ | 10 | 20.86 | 3 | 3 | #4 |
| ^[8an]^ | 1.2 | 4000 | 0.6 | 365 | #4 |
| ^[8ao]^ | 9 | 2.09 | 2 | 0.53 | #4 |
| ^[8ap]^ | 3 | 361.4 | 1.5 | 9.71 | #4 |
| ^[8aq]^ | 2.16 | 1068.28 | 1 | 28.64 | #4 |
| ^[8ar]^ | 3.7 | 5.4 | 1.25 | 2.05 | #4 |
| ^[8as]^ | 4 | 51.7 | 0.5 | 67.2 | #4 and see note #4 |
| ^[8at]^ | 0.9 | 653.4 | 0.5 | 64.99 | #4 |
| ^[8au]^ | 9.9 | 7.49 | 1.9 | 2.12 | #4 |
| ^[8av]^ | 0.03 | 635.9 | 0.01 | 196.4 | #4 |
| ^[8aw]^ | 14 | 12.84 | 2 | 3.64 | #4 |
| ^[8ax]^ | 7 | 1.62 | 2 | 1.07 | #4 |
| ^[8ay]^ | 0.7 | 1759 | 0.5 | 56 | #4 |
| ^[8az]^ | 2 | 11.35 | 0.6 | 1.54 | #4 |
| ^[8ba]^ | 5 | 6.82 | 1 | 1.79 | #4 |
| ^[9a]^ | 0.1 | 1214 | 0.03 | 146 | #4 |
| ^[9b]^ | 9 | 4.2 | 2 | 1.21 | #4 |
| ^[9c]^ | 9 | 10.9 | 3 | 2.05 | #4 |
| ^[9d]^ | 0.35 | 150000 | 0.0005 | 499 | #4 |
| ^[9e]^ | 8 | 4.43 | 2 | 1.70 | #4 |
| ^[9f]^ | 0.7 | 78.2 | 0.2 | 25 | #4 |
| ^[9g]^ | 4 | 7.16 | 1 | 2.55 | #4 |
| ^[9h]^ | 8 | 10.4 | 2 | 3.2 | #4 |
| ^[9i]^ | 8 | 15.56 | 1.1 | 1.75 | #4, see note #4 |
| ^[9j]^ | 0.45 | 2557.71 | 0.15 | 7.65 | #4 |
| ^[9k]^ | 3.5 | 4047.5 | 1.5 | 4.6 | #4 |
| ^[9l]^ | 3.35 | 2911 | 0.5 | 37.5 | #4 |
| ^[9m]^ | 12 | 4.5 | 2 | 1.45 | #4 |
| ^[9n]^ | 1.5 | 8732.41 | 0.2 | 412.56 | #4 |
| ^[9o]^ | 20 | 13.1 | 0.8 | 1.9 | #4 |
| ^[10a]^ | 5 | 983 | 0.6 | 1.8 | #4 |
| ^[10b]^ | 0.5 | 2408.955 | 0.1 | 49.579 | #4 |
| ^[10c]^ | 0.24 | 3854 | 0.07 | 101.6 | #4 |
| ^[10d]^ | 0.83 | 8787.4 | 0.15 | 256.1 | #4, see note #4 |
| ^[10e]^ | 5 | 11.35 | 1 | 3.87 | #4 |
| ^[11a]^ | 0.2 | 6.07 | 0.04 | 0.58 | #4 |
| ^[11b]^ | 4.85 | 63494 | 0.2 | 198 | #4 |
| ^[11c]^ | 0.3 | 6436.8 | 0.05 | 13 | #4 |
| ^[11d]^ | 2 | 264.01 | 0.4 | 5.36 | #4 |
| ^[11e]^ | 2 | 260000 | 0.2 | 33.1 | #4 |
| ^[12]^ | 2 | 1130 | 1.5 | 16.67 | #4 |
| ^[13]^ | N/A | N/A | 0.7 | 1.979 | N/A |
| ^[14]^ | N/A | N/A | 0.05 | 85.15 | See note 3 |
| ^[15]^ | N/A | N/A | 0.003 | 13 | N/A |
| ^[16]^ | N/A | N/A | 0.14 | 9 | N/A |
| ^[17]^ | N/A | N/A | 2 | 1.49 | See note #2 |
| ^[18]^ | N/A | N/A | 0.006 | 40.8 | N/A |
| ^[19]^ | N/A | N/A | 3 | 2 | N/A |
| ^[20]^ | N/A | N/A | 8 | 0.88 | See note #2 |
| ^[21]^ | N/A | N/A | 1 | 0.467 | N/A |
| ^[22]^ | N/A | N/A | 2 | 1.003 | See note #2 |
| ^[23]^ | N/A | N/A | 7 | 1.92 | See note #2 |
| ^[24]^ | N/A | N/A | 0.2 | 2.5 | N/A |
| ^[25]^ | N/A | N/A | 1 | 2.18 | See note #2 |
| ^[26]^ | N/A | N/A | 2 | 8.81 | N/A |
| ^[27]^ | N/A | N/A | 0.4 | 6.39 | N/A |
| ^[28]^ | 0.06 | 1190 | 0.01 | 673 | #4 |
| ^[29]^ | N/A | N/A | 0.03 | 54.58 | See note 3 |
| ^[30]^ | N/A | N/A | 0.25 | 15.3 | See note 2 |
| ^[31]^ | N/A | N/A | 0.2 | 58.5 | See note 2 |
| ^[32]^ | N/A | N/A | 0.01 | 5.67 | See note #3 |
| ^[33]^ | N/A | N/A | 0.01 | 107 | N/A |
| ^[34]^ | N/A | N/A | 0.05 | 20.8 | N/A |
| ^[35]^ | 2 | 0.73 | 0.035 | 66.32 | See note #3 |
| ^[36]^ | 0.1 | 501.6 | 0.06 | 142.5 | #2 and #4, see note #4 |
| ^[37]^ | N/A | N/A | 1 | 49.5 | N/A |
| ^[38]^ | N/A | N/A | 3.5 | 1.81 | N/A |
| ^[39]^ | N/A | N/A | 0.15 | 240 | See note #2 and #3 |
| ^[40]^ | 1.2 | 1665.9 | 0.8 | 35.2 | #4 |
| ^[41]^ | N/A | N/A | 0.01 | 387.8 | See note #3 |
| ^[42]^ | N/A | N/A | 0.016 | 111 | See note #4 |
| ^[43]^ | N/A | N/A | 0.2 | 153.02 | See note #3 |
| ^[44]^ | 0.282 | 67.2 | N/A | N/A | #2 |
| ^[45]^ | 3 | 6.78 | 1 | 2.98 | #4 |
| ^[46]^ | N/A | N/A | 0.01 | 6.5 | See note #3 |
| ^[47]^ | N/A | N/A | 1 | 2.98 | See note #4 |
| ^[48]^ | N/A | N/A | 1.8 | 2.2 | See note #4 |
| ^[49]^ | N/A | N/A | 0.1 | 0.69 | See note #4 |
| ^[50]^ | N/A | N/A | 0.005 | 201.8 | N/A |
| ^[51]^ | N/A | N/A | 0.25 | 14.108 | See note #2 |
| ^[52]^ | N/A | N/A | 0.025 | 35 | N/A |
| ^[53]^ | N/A | N/A | 0.002 | 146 | N/A |
| ^[54]^ | N/A | N/A | 6 | 1.596 | See note #4 |
| ^[55]^ | N/A | N/A | 0.1 | 10 | N/A |
| ^[56]^ | N/A | N/A | 0.3 | 92.45 | See note #4 |
| ^[57]^ | N/A | N/A | 0.1 | 494 | N/A |
| ^[58]^ | N/A | N/A | 2.3 | 0.9 | N/A |
| ^[59]^ | 1.3 | 21.4 | 0.7 | 7.3 | #4 |

**Table S1: Literary Data.** Values for maximum absolute reported gauge factor (|*G*_R_|), absolute reported maximum strain (|ε_max_|), absolute true gauge factor (|*G*_T_|) via Equation 1 fitting and absolute working factor (|*W*|) values tabulated. Also shown are the issues with gauge factor reporting that arise for each referenced study.

Notes

1. The low strain region for data quoting *G* = 45300 was not displayed. We note this discrepancy with gauge factor theory and use data from the work that is representative of the issue noted.
2. The value for *G* appears correct. However, the data sampling is very low and may result in Issue #3 factoring in.
3. Though the correct value is quoted in the abstract, a multi-fitting method akin to Issue #4 was still applied.
4. Low data sampling observed.

**References**

[1] a)Y. Deng, X. Guo, Y. Lin, Z. Huang, Y. Li, Dual-Phase Inspired Soft Electronic Sensors with Programmable and Tunable Mechanical Properties, *ACS Nano* **2023**, 17, 6423; b)J. Jia, Y. Peng, X.-J. Zha, K. Ke, R.-Y. Bao, Z.-Y. Liu, M.-B. Yang, W. Yang, Janus and Heteromodulus Elastomeric Fiber Mats Feature Regulable Stress Redistribution for Boosted Strain Sensing Performance, *ACS Nano* **2022**, 16, 16806; c)J. Gao, Y. Fan, Q. Zhang, L. Luo, X. Hu, Y. Li, J. Song, H. Jiang, X. Gao, L. Zheng, W. Zhao, Z. Wang, W. Ai, Y. Wei, Q. Lu, M. Xu, Y. Wang, W. Song, X. Wang, W. Huang, Ultra-Robust and Extensible Fibrous Mechanical Sensors for Wearable Smart Healthcare, *Advanced Materials* **2022**, 34, 2107511; d)J. Ma, X. Huo, J. Yin, S. Cai, K. Pang, Y. Liu, C. Gao, Z. Xu, Axially Encoded Mechano-Metafiber Electronics by Local Strain Engineering, *Advanced Materials* **2023**, 35, 2305615; e)J. Li, Q. Dai, Z. Wang, Y. Yi, Y. Shen, Z. Yao, S. Niu, Z. Han, L. Ren, Highly Robust and Self-Adhesive Soft Strain Gauge via Interface Design Engineering, *Advanced Materials* **2024**, 36, 2406432; f)G. Tian, D. Yang, C. Liang, Y. Liu, J. Chen, Q. Zhao, S. Tang, J. Huang, P. Xu, Z. Liu, D. Qi, A Nonswelling Hydrogel with Regenerable High Wet Tissue Adhesion for Bioelectronics, *Advanced Materials* **2023**, 35, 2212302; g)S. Mei, H. Yi, J. Zhao, Y. Xu, L. Shi, Y. Qin, Y. Jiang, J. Guo, Z. Li, L. Wu, High-density, highly sensitive sensor array of spiky carbon nanospheres for strain field mapping, *Nature Communications* **2024**, 15, 3752; h)Y. Zhao, C.-Y. Lo, L. Ruan, C.-H. Pi, C. Kim, Y. Alsaid, I. Frenkel, R. Rico, T.-C. Tsao, X. He, Somatosensory actuator based on stretchable conductive photothermally responsive hydrogel, *Science Robotics* **2021**, 6, eabd5483; i)M.-Y. Liu, C.-Z. Hang, X.-Y. Wu, L.-Y. Zhu, X.-H. Wen, Y. Wang, X.-F. Zhao, H.-L. Lu, Investigation of stretchable strain sensor based on CNT/AgNW applied in smart wearable devices, *Nanotechnology* **2022**, 33, 255501; j)S. Das, R. Singh, A. Das, S. Bag, R. P. Paily, U. Manna, Abrasion tolerant, non-stretchable and super-water-repellent conductive & ultrasensitive pattern for identifying slow, fast, weak and strong human motions under diverse conditions, *Mater Horiz* **2021**, 8, 2851; k)C. He, S. Sun, P. Wu, Intrinsically stretchable sheath-core ionic sensory fibers with well-regulated conformal and reprogrammable buckling, *Mater Horiz* **2021**, 8, 2088; l)Y. Zhang, Y. Zhang, W. Deng, Q. Li, M. Guo, G. Chen, Skin‐Like Soft Thermoelectric Composites with a “J‐Shaped” Stress–Strain Behavior for Self‐Powered Strain Sensing, *Advanced Functional Materials* **2024**, n/a, 2420644; m)H.-Q. Shao, K.-D. Wei, T. Gong, J. Jia, C.-Y. Tang, X.-J. Zha, K. Ke, R.-Y. Bao, K. Zhang, Y. Wang, W. Yang, Elastic Janus Microarray Film Strain Sensors with Heterogeneous Modulus and Conductivity for Healthcare and Braille Identification, *Advanced Functional Materials* **2024**, 34, 2316134; n)H. Li, H. Zheng, Y. J. Tan, S. B. Tor, K. Zhou, Development of an Ultrastretchable Double-Network Hydrogel for Flexible Strain Sensors, *ACS applied materials & interfaces* **2021**, 13, 12814; o)S. J. Paul, I. Elizabeth, S. Srivastava, J. S. Tawale, P. Chandra, H. C. Barshilia, B. K. Gupta, Epidermal Inspired Flexible Sensor with Buckypaper/PDMS Interfaces for Multimodal and Human Motion Monitoring Applications, *ACS Omega* **2022**, 7, 37674; p)J. Jia, Y. Peng, K. Ke, Z.-Y. Liu, W. Yang, Achieving a Wide-Range Linear Piezoresistive Response in Electrowritten Soft–Hard Polymer Blends via Salami-Inspired Heterostructure Design, *ACS applied materials & interfaces* **2024**, 16, 7939; q)X. Qing, Z. Liu, A. Katsaounis, N. Bouropoulos, I. Taurino, P. Fardim, Poly(vinyl alcohol)/Pullulan/NaCl Conductive Hydrogels with High Strength and Sensitivity for Wearable Strain Sensors, *ACS Appl. Polym. Mater.* **2024**, 6, 8105; r)K. Chang, T. Liu, Highly Stretchable Strain Sensor with Both an Ultrawide Workable Range and an Ultralow Detection Limit for Human Health Monitoring, *ACS Appl. Nano Mater.* **2024**, 7, 24697; s)Y. Zhang, C. Li, B. Zhou, H. He, Y. Zhou, L. Jiang, F.-L. Zhou, S. Chen, A flexible strain sensor based on conductive TPU/CNTs-Gr composites, *J. Appl. Polym. Sci.* **2022**, 139, e52475; t)T. Yu, X. Lü, W. Bao, High electrical self-healing flexible strain sensor based on MWCNT- polydimethylsiloxane elastomer with high gauge factor and wide measurement range, *Composites Science and Technology* **2023**, 238, 110049; u)L. Ara, M. Sher, M. Khan, T. U. Rehman, L. A. Shah, H.-M. Yoo, Dually-crosslinked ionic conductive hydrogels reinforced through biopolymer gellan gum for flexible sensors to monitor human activities, *International Journal of Biological Macromolecules* **2024**, 276, 133789; v)A. del Bosque, X. F. Sánchez-Romate, F. Cadini, C. Sbarufatti, M. Sánchez, M. Giglio, A. Ureña, Dual effect of temperature and strain on the electrical response of highly sensitive silicone elastomers doped with graphene nanoplatelets, *Polymer* **2023**, 286, 126419; w)H. M. Soe, R. Kumar, A. Matsuda, M. Mariatti, Surface-modification of silver nanoparticle–based polydimethylsiloxane composite for fabrication of strain sensor, *Materials Today Communications* **2023**, 36, 106486.

[2] a)H. He, T. Yang, T. Liu, Y. Gao, Z. Zhang, Z. Yang, F. Liang, Soft-Hard Janus Nanoparticles Triggered Hierarchical Conductors with Large Stretchability, High Sensitivity, and Superior Mechanical Properties, *Advanced Materials* **2024**, 36, 2312278; b)Y. Peng, H. Peng, Z. Chen, J. Zhang, Ultrasensitive Soft Sensor from Anisotropic Conductive Biphasic Liquid Metal-Polymer Gels, *Advanced Materials* **2024**, 36, 2305707; c)Q. Liu, X. Dong, H. Qi, H. Zhang, T. Li, Y. Zhao, G. Li, W. Zhai, 3D printable strong and tough composite organo-hydrogels inspired by natural hierarchical composite design principles, *Nature Communications* **2024**, 15, 3237; d)C. Muhammed Ajmal, S. Cha, W. Kim, K. P. Faseela, H. Yang, S. Baik, Invariable resistance of conductive nanocomposite over 30% strain, *Science Advances* **2022**, 8, eabn3365; e)Y. Kato, K. Fukuda, T. Someya, T. Yokota, An ultra-flexible temperature-insensitive strain sensor, *Journal of Materials Chemistry C* **2023**, 11, 14070; f)N. Qaiser, F. Al-Modaf, S. M. Khan, S. F. Shaikh, N. El-Atab, M. M. Hussain, A Robust Wearable Point-of-Care CNT-Based Strain Sensor for Wirelessly Monitoring Throat-Related Illnesses, *Advanced Functional Materials* **2021**, 31, 2103375; g)Y. Feng, C. Wu, M. Chen, H. Sun, A. L. R. Vellaisamy, W. A. Daoud, X. Yu, G. Zhang, W. J. Li, Amoeba-Inspired Self-Healing Electronic Slime for Adaptable, Durable Epidermal Wearable Electronics, *Advanced Functional Materials* **2024**, 34, 2402393; h)X. Bian, Z. Yang, T. Zhang, J. Yu, G. Xu, A. Chen, Q. He, J. Pan, Multifunctional Flexible AgNW/MXene/PDMS Composite Films for Efficient Electromagnetic Interference Shielding and Strain Sensing, *ACS applied materials & interfaces* **2023**, 15, 41906; i)C. Yang, W. Huang, Y. Lin, S. Cao, H. Wang, Y. Sun, T. Fang, M. Wang, D. Kong, Stretchable MXene/Carbon Nanotube Bilayer Strain Sensors with Tunable Sensitivity and Working Ranges, *ACS applied materials & interfaces* **2024**, 16, 30274; j)W. Yao, Y. Yan, J. Sun, Z. Zhang, W. Sun, W. Huang, J. Cheng, H. Zhao, M. Xie, Q. Sun, G. Huang, X. Lin, Mechanically Durable Superhydrophobic Strain Sensors with High Biocompatibility and Sensing Performance for Underwater Motion Monitoring, *ACS applied materials & interfaces* **2024**, 16, 6548; k)Y. He, S. Sun, X. Zhang, Y. Xu, C. Zhang, C. Shao, J. Yang, J. Wen, Self-Adhesive, Anti-Freezing Multifunctional Zwitterionic Hydrogels with Lignin-Promoted Rapid Gelation for Flexible Strain Sensors, *Acs Sustain Chem Eng* **2024**, 12, 11809; l)D. Mai, J. Mo, S. Shan, Y. Lin, A. Zhang, Self-Healing, Self-Adhesive Strain Sensors Made with Carbon Nanotubes/Polysiloxanes Based on Unsaturated Carboxyl–Amine Ionic Interactions, *ACS applied materials & interfaces* **2021**, 13, 49266; m)L. Zhao, J. Qiao, F. Li, D. Yuan, J. Huang, M. Wang, S. Xu, Laser-Patterned Hierarchical Aligned Micro-/Nanowire Network for Highly Sensitive Multidimensional Strain Sensor, *ACS applied materials & interfaces* **2022**, 14, 48276; n)Z. Jin, H. Zhou, J. Lai, X. Jin, H. Liu, P. Wu, W. Chen, A. Ma, Self-Recoverable, Stretchable, and Sensitive Wearable Sensors Based on Ternary Semi-interpenetrating Ionic Hydrogels, *ACS Appl. Polym. Mater.* **2021**, 3, 2732; o)W. Zhao, Z. Lin, Z. Sun, Z. Zhu, W. Lin, Y. Xu, Z. Peng, Z. Sun, Z. Wang, Road Narrow-Inspired Strain Concentration to Wide-Range-Tunable Gauge Factor of Ionic Hydrogel Strain Sensor, *Advanced Science* **2023**, 10, 2303338; p)Z. Chen, J. Sun, Z. Zhan, Y. Yuan, X. Tian, J. Jin, W. Wu, K. Ayikanbaier, Multifunctional flexible strain sensor based on three-dimensional core-shell structures of silver nanoparticles/natural rubber, *J. Appl. Polym. Sci.* **2024**, 141, e55436; q)J. Li, D. Xiang, C. Zhao, H. Li, L. Zhou, L. Wang, G. Yan, Z. Li, P. Wang, B. Wang, Y. Wu, Flexible strain sensors with high sensitivity and large monitoring range prepared by biaxially stretching conductive polymer composites with a bilayer structure, *J. Appl. Polym. Sci.* **2023**, 140, e54718; r)W. Muhammad, S.-D. Kim, Highly sensitive and flexible micro-patterned PPy/PDMS strain sensors with enhanced conductivity and stretchability for wearable electronics, *Polymer* **2024**, 308, 127356.

[3] a)S. P. Ogilvie, M. J. Large, M. A. O’Mara, A. C. Sehnal, A. Amorim Graf, P. J. Lynch, A. J. Cass, J. P. Salvage, M. Alfonso, P. Poulin, A. A. K. King, A. B. Dalton, Nanosheet-Stabilized Emulsions: Near-Minimum Loading and Surface Energy Design of Conductive Networks, *ACS Nano* **2022**, 16, 1963; b)Y. Choi, K. Kang, D. Son, M. Shin, Molecular Rationale for the Design of Instantaneous, Strain-Tolerant Polymeric Adhesive in a Stretchable Underwater Human–Machine Interface, *ACS Nano* **2022**, 16, 1368; c)Z. Wang, R. Xue, H. Zhang, Y. Zhang, X. Tang, H. Wang, A. Shao, Y. Ma, A Hydrogel Electrolyte toward a Flexible Zinc-Ion Battery and Multifunctional Health Monitoring Electronics, *ACS Nano* **2024**, 18, 7596; d)L. Wang, X. Xu, J. Chen, W. Su, F. Zhang, A. Li, C. Li, C. Xu, Y. Sun, Crack Sensing of Cardiomyocyte Contractility with High Sensitivity and Stability, *ACS Nano* **2022**, 16, 12645; e)Y. Cai, J. Shen, J.-H. Fu, N. Qaiser, C. Chen, C.-C. Tseng, M. Hakami, Z. Yang, H.-J. Yen, X. Dong, L.-J. Li, Y. Han, V. Tung, Graphdiyne-Based Nanofilms for Compliant On-Skin Sensing, *ACS Nano* **2022**, 16, 16677; f)H. Yang, J. Li, X. Xiao, J. Wang, Y. Li, K. Li, Z. Li, H. Yang, Q. Wang, J. Yang, J. S. Ho, P.-L. Yeh, K. Mouthaan, X. Wang, S. Shah, P.-Y. Chen, Topographic design in wearable MXene sensors with in-sensor machine learning for full-body avatar reconstruction, *Nature Communications* **2022**, 13, 5311; g)H. Yang, S. Ding, J. Wang, S. Sun, R. Swaminathan, S. W. L. Ng, X. Pan, G. W. Ho, Computational design of ultra-robust strain sensors for soft robot perception and autonomy, *Nature Communications* **2024**, 15, 1636; h)S. Wu, S. Ma, M. Liu, Z. Jiang, Y. Zhou, S. Chen, L. Jiang, Silver nanoparticles coated microcracked poly(styrene butadiene styrene)/graphene/carbon nanotube composite fiber for high performance strain sensing, *Eur Polym J* **2024**, 214, 113182.

[4] a)Y. Luo, X. Chen, X. Li, H. Tian, S. Li, L. Wang, J. He, Z. Yang, J. Shao, Heterogeneous Strain Distribution Based Programmable Gated Microchannel for Ultrasensitive and Stable Strain Sensing, *Advanced Materials* **2023**, 35, 2207141; b)H. Liu, C. Du, L. Liao, H. Zhang, H. Zhou, W. Zhou, T. Ren, Z. Sun, Y. Lu, Z. Nie, F. Xu, J. Zhu, W. Huang, Approaching intrinsic dynamics of MXenes hybrid hydrogel for 3D printed multimodal intelligent devices with ultrahigh superelasticity and temperature sensitivity, *Nature Communications* **2022**, 13, 3420; c)Q. Lyu, S. Gong, J. G. Lees, J. Yin, L. W. Yap, A. M. Kong, Q. Shi, R. Fu, Q. Zhu, A. Dyer, J. M. Dyson, S. Y. Lim, W. Cheng, A soft and ultrasensitive force sensing diaphragm for probing cardiac organoids instantaneously and wirelessly, *Nature Communications* **2022**, 13, 7259; d)N. Wang, X. Yang, X. Zhang, Ultrarobust subzero healable materials enabled by polyphenol nano-assemblies, *Nature Communications* **2023**, 14, 814; e)D. Bai, F. Liu, D. Xie, F. Lv, L. Shen, Z. Tian, 3D printing of flexible strain sensor based on MWCNTs/flexible resin composite, *Nanotechnology* **2022**, 34, 045701; f)J. Li, S. Li, Y. Su, Stretchable Strain Sensors Based on Deterministic-Contact-Resistance Braided Structures with High Performance and Capability of Continuous Production, *Advanced Functional Materials* **2022**, 32, 2208216; g)Z. Lu, J. Wang, L. He, J. Song, Z. Yang, F. A. Hammad, High-Performance Multidirectional Flexible Strain Sensor for Human Motion and Health Monitoring, *ACS applied materials & interfaces* **2024**, 16, 41409; h)J. Seong, J. H. Lee, M. W. Han, Soft Sensors via Conductive Textile Stitching: Enabling Strain, Tactile, and Volumetric Sensing, *Advanced Materials Technologies* **2024**, n/a, 2401306; i)L.-Q. Tao, C. Gao, G. Wang, H. Sun, L.-Y. Guo, T.-L. Ren, High sensitivity graphene based health sensor with self-warning function, *Composites Science and Technology* **2023**, 241, 110123; j)L. Wang, C. Wu, C. Li, S. Ji, X. Peng, R. Cao, Wavy graphene mesh reinforced elastic composite films for strain and pressure sensing, *Mater Lett* **2023**, 349, 134862; k)M. Mardani, S. Hossein Hosseini Lavassani, M. Adresi, A. Rashidi, Piezoresistivity and mechanical properties of self-sensing CNT cementitious nanocomposites: Optimizing the effects of CNT dispersion and surfactants, *Construction and Building Materials* **2022**, 349, 128127.

[5] a)J. Xu, X. Li, H. Chang, B. Zhao, X. Tan, Y. Yang, H. Tian, S. Zhang, T.-L. Ren, Electrooculography and Tactile Perception Collaborative Interface for 3D Human–Machine Interaction, *ACS Nano* **2022**, 16, 6687; b)J. H. Lee, S. H. Kim, J. S. Heo, J. Y. Kwak, C. W. Park, I. Kim, M. Lee, H.-H. Park, Y.-H. Kim, S. J. Lee, S. K. Park, Heterogeneous Structure Omnidirectional Strain Sensor Arrays With Cognitively Learned Neural Networks, *Advanced Materials* **2023**, 35, 2208184; c)A. Roy, S. Zenker, S. Jain, R. Afshari, Y. Oz, Y. Zheng, N. Annabi, A Highly Stretchable, Conductive, and Transparent Bioadhesive Hydrogel as a Flexible Sensor for Enhanced Real-Time Human Health Monitoring, *Advanced Materials* **2024**, 36, 2404225; d)S. Peng, S. Wu, Y. Yu, Z. Sha, G. Li, T. T. Hoang, M. T. Thai, T. N. Do, D. Chu, C. H. Wang, Carbon nanofiber-reinforced strain sensors with high breathability and anisotropic sensitivity, *J Mater Chem A* **2021**, 9, 26788; e)J. Zhang, Y. Liang, Z. Deng, H. Xu, H. Zhang, B. Guo, J. Zhang, Adhesive Ion-Conducting Hydrogel Strain Sensor with High Sensitivity, Long-Term Stability, and Extreme Temperature Tolerance, *ACS applied materials & interfaces* **2023**, 15, 29902; f)D. Wei, M. Xu, A. Zhang, Y. Ouyang, X. Zheng, Y. Shi, X. Li, Q. Zhang, X. Zhang, Wide Linear Range Strain Sensor Enabled by the Non-Newtonian Fluid for Bio-Signals Monitoring, *Advanced Engineering Materials* **2022**, 24, 2200100.

[6] a)Q. Wu, Y. Xu, S. Han, A. Chen, J. Zhang, Y. Chen, X. Yang, L. Guan, Versatile Hydrogel Based on a Controlled Microphase-Separation Strategy for Both Liquid- and Solid-Phase 3D Printing, *ACS Nano* **2024**, 18, 31148; b)M. Lei, K. Feng, S. Ding, M. Wang, Z. Dai, R. Liu, Y. Gao, Y. Zhou, Q. Xu, B. Zhou, Breathable and Waterproof Electronic Skin with Three-Dimensional Architecture for Pressure and Strain Sensing in Nonoverlapping Mode, *ACS Nano* **2022**, 16, 12620; c)X.-Q. Wang, K. H. Chan, W. Lu, T. Ding, S. W. L. Ng, Y. Cheng, T. Li, M. Hong, B. C. K. Tee, G. W. Ho, Macromolecule conformational shaping for extreme mechanical programming of polymorphic hydrogel fibers, *Nature Communications* **2022**, 13, 3369; d)M. Sun, H. Li, Y. Hou, N. Huang, X. Xia, H. Zhu, Q. Xu, Y. Lin, L. Xu, Multifunctional tendon-mimetic hydrogels, *Science Advances* **2023**, 9, eade6973; e)X. Cui, J. Guo, S. Araby, F. Abbassi, C. Zhang, A. L. Diaby, Q. Meng, Porous polyvinyl alcohol/graphene oxide composite film for strain sensing and energy-storage applications, *Nanotechnology* **2022**, 33, 415701; f)Y. Wang, W. Qin, M. Yang, Z. Tian, W. Guo, J. Sun, X. Zhou, B. Fei, B. An, R. Sun, S. Yin, Z. Liu, High Linearity, Low Hysteresis Ti3C2T MXene/AgNW/Liquid Metal Self-Healing Strain Sensor Modulated by Dynamic Disulfide and Hydrogen Bonds, *Advanced Functional Materials* **2023**, 33, 2301587; g)H. Shi, H. Huo, H. Yang, H. Li, J. Shen, J. Wan, G. Du, L. Yang, Cellulose-Based Dual-Network Conductive Hydrogel with Exceptional Adhesion, *Advanced Functional Materials* **2024**, 34, 2408560.

[7] a)D. Yang, H. K. Nam, T.-S. D. Le, J. Yeo, Y. Lee, Y.-R. Kim, S.-W. Kim, H.-J. Choi, H. C. Shim, S. Ryu, S. Kwon, Y.-J. Kim, Multimodal E-Textile Enabled by One-Step Maskless Patterning of Femtosecond-Laser-Induced Graphene on Nonwoven, Knit, and Woven Textiles, *ACS Nano* **2023**, 17, 18893; b)L. Luo, Z. Wu, Q. Ding, H. Wang, Y. Luo, J. Yu, H. Guo, K. Tao, S. Zhang, F. Huo, J. Wu, In Situ Structural Densification of Hydrogel Network and Its Interface with Electrodes for High-Performance Multimodal Artificial Skin, *ACS Nano* **2024**, 18, 15754; c)K. Park, S. An, J. Kim, S. Yoon, J. Song, D. Jung, J. Park, Y. Lee, D. Son, J. Seo, Resealable Antithrombotic Artificial Vascular Graft Integrated with a Self-Healing Blood Flow Sensor, *ACS Nano* **2023**, 17, 7296; d)Y. Yang, N. Wu, B. Li, W. Liu, F. Pan, Z. Zeng, J. Liu, Biomimetic Porous MXene Sediment-Based Hydrogel for High-Performance and Multifunctional Electromagnetic Interference Shielding, *ACS Nano* **2022**, 16, 15042; e)C. Dang, Y. Shao, S. Ding, H. Qi, W. Zhai, Polyfunctional and Multisensory Bio-Ionoelastomers Enabled by Covalent Adaptive Networks With Hierarchically Dynamic Bonding, *Advanced Materials* **2024**, 36, 2406967; f)X. Fan, Y. Luo, K. Li, Y. J. Wong, C. Wang, J. C. C. Yeo, G. Yang, J. Li, X. J. Loh, Z. Li, X. Chen, A Recyclable Ionogel with High Mechanical Robustness Based on Covalent Adaptable Networks, *Advanced Materials* **2024**, 36, 2407398; g)L. Cheng, C. S. Yeung, L. Huang, G. Ye, J. Yan, W. Li, C. Yiu, F.-R. Chen, H. Shen, B. Z. Tang, Y. Ren, X. Yu, R. Ye, Flash healing of laser-induced graphene, *Nature Communications* **2024**, 15, 2925; h)Y. Zhang, J. Yang, X. Hou, G. Li, L. Wang, N. Bai, M. Cai, L. Zhao, Y. Wang, J. Zhang, K. Chen, X. Wu, C. Yang, Y. Dai, Z. Zhang, C. F. Guo, Highly stable flexible pressure sensors with a quasi-homogeneous composition and interlinked interfaces, *Nature Communications* **2022**, 13, 1317; i)K. Zhang, W. Jiang, X. Li, X. Gao, Highly stretchable and sensitive strain sensors based on modified PDMS and hybrid particles of AgNWs/graphene, *Nanotechnology* **2022**, 34, 06LT01; j)X. Song, X. Liu, Y. Peng, Z. Xu, W. Liu, K. Pang, J. Wang, L. Zhong, Q. Yang, J. Meng, A graphene-coated silk-spandex fabric strain sensor for human movement monitoring and recognition, *Nanotechnology* **2021**, 32, 215501; k)F. Luo, Y. Qin, X. Wang, X. Zhao, K. Chen, W. Huang, Enhanced high-strength, temperature-resistant PVA hydrogel sensors with silica/xanthan/glycerol for posture monitoring and handwriting recognition using deep learning, *Journal of Materials Chemistry C* **2024**, 12, 14844; l)X. Di, J. Li, M. Yang, Q. Zhao, G. Wu, P. Sun, Bioinspired, nucleobase-driven, highly resilient, and fast-responsive antifreeze ionic conductive hydrogels for durable pressure and strain sensors, *J Mater Chem A* **2021**, 9, 20703; m)Z. Xie, F. Meng, J. Yang, Y. Wang, C. B. Park, P. Gong, G. Li, High sensing performance flexible nanocomposite sensor with a hybrid nanostructure constructed via nanoscale confined motion of nanofibers and nanoplatelets, *Nanoscale* **2024**, 16, 20288; n)H. Zhou, J. Lai, B. Zheng, X. Jin, G. Zhao, H. Liu, W. Chen, A. Ma, X. Li, Y. Wu, From Glutinous-Rice-Inspired Adhesive Organohydrogels to Flexible Electronic Devices Toward Wearable Sensing, Power Supply, and Energy Storage, *Advanced Functional Materials* **2022**, 32, 2108423; o)D. Yang, H. K. Nam, Y. Lee, S. Kwon, J. Lee, H. Yoon, Y.-J. Kim, Laser-Induced Graphene Smart Textiles for Future Space Suits and Telescopes, *Advanced Functional Materials* **2025**, 35, 2411257; p)L. Yi, Y. Zhao, Y. Li, D. Guo, Z. Zeng, Z. Liu, H. Zheng, G. J. Cheng, F. Liu, Intelligent Cobweb Structures for All‐In‐One Flexible Devices via Laser Thermal Printing, *Advanced Functional Materials* **2024**, n/a, 2413256; q)X. Lu, Y. Si, S. Zhang, J. Yu, B. Ding, In Situ Synthesis of Mechanically Robust, Transparent Nanofiber-Reinforced Hydrogels for Highly Sensitive Multiple Sensing, *Advanced Functional Materials* **2021**, 31, 2103117; r)J. Pu, Y. Gao, Z. Geng, Y. Zhang, Q. Cao, J. Yang, X. Zhao, Y. Wang, J. Wang, C. Guan, Grafted MXene Assisted Bifunctional Hydrogel for Stable and Highly Sensitive Self-Powered Fibrous System, *Advanced Functional Materials* **2024**, 34, 2304453; s)A. Maji, C. Kuila, U. Phadikar, N. C. Murmu, T. Kuila, Sustainable Engineering of the Wearable Sensor for Noninvasive Health Monitoring Using Exfoliated Layered Double Hydroxide/Reduced Graphene Oxide/Poly(vinyl alcohol) Electrospun Fiber Composites, *ACS Appl. Polym. Mater.* **2024**, 6, 13002; t)H. Wang, J. Liu, H. Cui, Y. Liu, J. Zhu, H. Wang, G. Song, Z. Li, D. Chen, Strain Sensor with High Sensitivity and Large Response Range Based on Self-Assembled Elastic-Sliding Conductive Networks, *ACS Appl. Electron. Mater.* **2021**, 3, 1758; u)I. V. Novikov, D. V. Krasnikov, A. M. Vorobei, Y. I. Zuev, H. A. Butt, F. S. Fedorov, S. A. Gusev, A. A. Safonov, E. V. Shulga, S. D. Konev, I. V. Sergeichev, S. S. Zhukov, T. Kallio, B. P. Gorshunov, O. O. Parenago, A. G. Nasibulin, Multifunctional Elastic Nanocomposites with Extremely Low Concentrations of Single-Walled Carbon Nanotubes, *ACS applied materials & interfaces* **2022**, 14, 18866; v)Q. Ling, T. Ke, W. Liu, Z. Ren, L. Zhao, H. Gu, Tough, Repeatedly Adhesive, Cyclic Compression-Stable, and Conductive Dual-Network Hydrogel Sensors for Human Health Monitoring, *Ind. Eng. Chem. Res.* **2021**, 60, 18373; w)W. Pan, L. Xu, S. C. Lamont, Y. Zhang, J. Ding, F. J. Vernerey, Thermosensitive MXene-Based Flexible Wearable Sensors for Multifunctional Human Signals Monitoring, *ACS Appl. Polym. Mater.* **2024**, 6, 9488; x)Q. Cao, Z. Shu, T. Zhang, W. Ji, J. Chen, Y. Wei, Highly Elastic, Sensitive, Stretchable, and Skin-Inspired Conductive Sodium Alginate/Polyacrylamide/Gallium Composite Hydrogel with Toughness as a Flexible Strain Sensor, *Biomacromolecules* **2022**, 23, 2603; y)L. Ma, X. Lei, X. Guo, L. Wang, S. Li, T. Shu, G. J. Cheng, F. Liu, Carbon Black/Graphene Nanosheet Composites for Three-Dimensional Flexible Piezoresistive Sensors, *ACS Appl. Nano Mater.* **2022**, 5, 7142; z)A. T. Khedewy, D. I. Saleh, A. Shaker, On the Effect of Fibre Orientation and MWCNTs on the Strain-Sensing Performance of TPU/PANI Electrospun Nanofibres, *Polymers for Advanced Technologies* **2024**, 35, e6639; aa)L. Xiao, C. Ou, D. Zhang, Y. Ma, Z. Xu, Y. Zhou, H. Jiang, Strong Tough Poly Acrylic-co-acrylamide Hydrogels via a Synergistic Effect of Fiber and Metal-Ligand Bonds as Flexible Strain Sensors, *Macromol. Mater. Eng.* **2022**, 307, 2200389; ab)T. Song, M. Xu, Y. Weng, W. Zhang, Y. Shi, X. Li, Q. Zhang, Silkworm‐Shaped MoS2 Growing on Graphene Foam for Highly Sensitive and Flexible Strain Sensor with Full‐Scale Human Motion Detection Ability, *Advanced Materials Technologies* **2024**, n/a, 2401621; ac)X. Cheng, X. Cao, Z. Wu, Z. Ying, D. Camilleri, X. Hu, A Flexible Conformal Piezoresistive Sensor Based on Electrospinning for Deformation Monitoring of Carbon Fiber-Reinforced Polymer, *Advanced Engineering Materials* **2023**, 25, 2300341; ad)H. Li, L. Li, J. Wei, T. Chen, P. Wei, Salt-Adaptively Conductive Ionogel Sensor for Marine Sensing, *Small* **2024**, 20, 2305848; ae)Y. Gao, J. Sun, X. Tian, Y. Yuan, Stretchable Polyaniline@Epoxidized Natural Rubber Composites with Strong 3D Conductive Networks for High Performance Strain Sensors, *Macromol. Chem. Phys.* **2023**, 224, 2200365; af)X.-D. Li, H.-X. Huang, Facile fabrication of flexible TPU-based microcellular nanocomposite piezoresistive sensors with tunable piezoresistivity via modulating cell structure, *Polym Eng Sci* **2023**, 63, 1678; ag)J. Chen, B. Li, X. Ma, S. Zhou, Q. Gu, H. Bian, Z. Luo, Modified lignin-induced composite hydrogels with good mechanical properties, adhesion, and UV resistance for strain sensors, *J. Appl. Polym. Sci.* **2023**, 140, e54643; ah)S. Duan, Y. Wei, Y. Wang, L. Zhai, Y. Qin, Z. Guo, D. Li, W. Hou, S. Liu, X. Li, B. Zhu, P. Pan, M. Xu, J. Liu, H. Guo, Z. Xu, H. Tian, Y. Yang, T.-L. Ren, Giant gauge factors in an anchored sandwich structure with a soft break mechanism, *Cell Reports Physical Science* **2024**, 5, 101893; ai)K. Wang, Z. Wu, R. Wu, J. Zang, B. Lu, C. Du, Y. Yu, Direct fabrication of flexible strain sensor with adjustable gauge factor on medical catheters, *Journal of Science: Advanced Materials and Devices* **2023**, 8, 100558; aj)S. Li, W. Wu, Y. Chang, W. Chen, Y. Liu, Z. He, Y. Pu, I. S. Babichuk, T. T. Ye, Z. Gao, J. Yang, Flexible strain sensors based on silver nanowires and UV-curable acrylate elastomers for wrist movement monitoring, *RSC Applied Interfaces* **2024**, 1, 684; ak)N. Ding, Y. Bai, Y. Feng, X. Zou, Y. Chen, S. Bi, S. Liu, W. Zhao, Q. Zhao, Multifunctional MXene/PAA organohydrogel as a flexible strain sensor for wearable human–machine interaction, *RSC Appl. Polym.* **2023**, 1, 64; al)X. Guo, Y. Li, Z. Zeng, Y. Zhao, X. Lei, Y. Wang, D. Guo, F. Liu, Ultra-sensitive flexible pressure sensor with hierarchical structural laser-induced carbon nanosheets/carbon nanotubes composite film, *Composites Science and Technology* **2023**, 244, 110290; am)J. Yang, Y. Feng, B. Wang, J. Miao, S. Wei, H. Li, L. Mo, Z. Qin, Tough, multifunctional, and green double-network binary solvent eutectogel with in-situ generation of lignin nanoparticles based on one-step dual phase separations for wearable flexible strain sensors, *Chemical Engineering Journal* **2023**, 474, 145544; an)A. del Bosque, X. F. Sánchez-Romate, A. Gómez, M. Sánchez, A. Ureña, Highly stretchable strain sensors based on graphene nanoplatelet-doped ecoflex for biomedical purposes, *Sensors and Actuators A: Physical* **2023**, 353, 114249.

[8] a)T. Hu, T. Pan, D. Guo, Y. Xiao, F. Li, M. Gao, Z. Huang, J. Zhu, T. Cheng, Y. Lin, Omnidirectional Configuration of Stretchable Strain Sensor Enabled by the Strain Engineering with Chiral Auxetic Metamaterial, *ACS Nano* **2023**, 17, 22035; b)M. Zhu, W. Xu, L. Chen, D. Wu, Z. Wang, X. Hu, X. Luo, R. Xiong, C. Huang, Ultrathin Self-Healing Nanofibrous Membrane with a Hierarchical Confined Structure for Biomimetic Epidermal Electrodes, *ACS Nano* **2024**, 18, 28834; c)C. Liu, Y. Wang, S. Shi, Y. Zheng, Z. Ye, J. Liao, Q. Sun, B. Dang, X. Shen, Myelin Sheath-Inspired Hydrogel Electrode for Artificial Skin and Physiological Monitoring, *ACS Nano* **2024**, 18, 27420; d)L. Liu, R. Li, F. Liu, L. Huang, W. Liu, J. Wang, Z. Wu, N. Reddy, W. Cui, Q. Jiang, Highly Elastic and Strain Sensing Corn Protein Electrospun Fibers for Monitoring of Wound Healing, *ACS Nano* **2023**, 17, 9600; e)Q. Yang, N. Liu, J. Yin, H. Tian, Y. Yang, T.-L. Ren, Understanding the Origin of Tensile Response in a Graphene Textile Strain Sensor with Negative Differential Resistance, *ACS Nano* **2022**, 16, 14230; f)H. Wang, Z. Xiang, P. Zhao, J. Wan, L. Miao, H. Guo, C. Xu, W. Zhao, M. Han, H. Zhang, Double-Sided Wearable Multifunctional Sensing System with Anti-interference Design for Human–Ambience Interface, *ACS Nano* **2022**, 16, 14679; g)H. Tang, Y. Li, B. Chen, X. Chen, Y. Han, M. Guo, H.-q. Xia, R. Song, X. Zhang, J. Zhou, In Situ Forming Epidermal Bioelectronics for Daily Monitoring and Comprehensive Exercise, *ACS Nano* **2022**, 16, 17931; h)F. Chen, Q. Zhuang, Y. Ding, C. Zhang, X. Song, Z. Chen, Y. Zhang, Q. Mei, X. Zhao, Q. Huang, Z. Zheng, Wet-Adaptive Electronic Skin, *Advanced Materials* **2023**, 35, 2305630; i)R. Hu, X. Yang, W. Cui, L. Leng, X. Zhao, G. Ji, J. Zhao, Q. Zhu, J. Zheng, An Ultrahighly Stretchable and Recyclable Starch-Based Gel with Multiple Functions, *Advanced Materials* **2023**, 35, 2303632; j)B. Feng, T. Sun, W. Wang, Y. Xiao, J. Huo, Z. Deng, G. Bian, Y. Wu, G. Zou, W. Wang, T. Ren, L. Liu, Venation-Mimicking, Ultrastretchable, Room-Temperature-Attachable Metal Tapes for Integrated Electronic Skins, *Advanced Materials* **2023**, 35, 2208568; k)H. Jiang, B. Yuan, H. Guo, F. Pan, F. Meng, Y. Wu, X. Wang, L. Ruan, S. Zheng, Y. Yang, Z. Xiu, L. Li, C. Wu, Y. Gong, M. Yang, W. Lu, Malleable, printable, bondable, and highly conductive MXene/liquid metal plasticine with improved wettability, *Nature Communications* **2024**, 15, 6138; l)Y. Zhang, Y. Wang, Y. Guan, Y. Zhang, Peptide-enhanced tough, resilient and adhesive eutectogels for highly reliable strain/pressure sensing under extreme conditions, *Nature Communications* **2022**, 13, 6671; m)L. Chen, G. Chen, L. Bi, Z. Yang, Z. Wu, M. Huang, J. Bao, W. Wang, C. Ye, J. Pan, Y. Peng, C. Ye, A highly sensitive strain sensor with a sandwich structure composed of two silver nanoparticles layers and one silver nanowires layer for human motion detection, *Nanotechnology* **2021**, 32, 375504; n)A. Akouros, N. Koutroumanis, A. C. Manikas, G. Paterakis, M. G. P. Carbone, G. Anagnostopoulos, M. Dimitropoulos, C. Galiotis, Highly stretchable strain sensors based on Marangoni self-assemblies of graphene and its hybrids with other 2D materials, *Nanotechnology* **2023**, 34, 295501; o)H. Lu, Y. Feng, S. Wang, J. Liu, Q. Han, Q. Meng, A high-performance, sensitive, low-cost LIG/PDMS strain sensor for impact damage monitoring and localization in composite structures, *Nanotechnology* **2024**, 35, 355702; p)S. Yang, Y. Ling, Q. Wu, H. Zhang, Z. Yan, G. Huang, J. Lin, C. Wan, Lignin-derived porous graphene for wearable and ultrasensitive strain sensors, *Journal of Materials Chemistry C* **2022**, 10, 11730; q)Y. Liu, X. Fan, W. Feng, X. Shi, F. Li, J. Wu, X. Ji, J. Liang, An in situ and rapid self-healing strategy enabling a stretchable nanocomposite with extremely durable and highly sensitive sensing features, *Mater Horiz* **2021**, 8, 250; r)C. Luo, X. Deng, S. Xie, Design and performance of an ultra-sensitive and super-stretchable hydrogel for artificial skin, *Journal of Materials Chemistry C* **2021**, 9, 17042; s)C. Yang, D. Zhang, D. Wang, X. Chen, H. Luan, Ultra-sensitive, stretchable, and bidirectional wearable strain sensor for human motion detection, *Journal of Materials Chemistry C* **2022**, 10, 7076; t)Y. Wang, Y. Shuang, M. Kim, D. Ando, F. Narita, Y. Sutou, An amorphous Cr2Ge2Te6/polyimide double-layer foil with an extraordinarily outstanding strain sensing ability, *Mater Horiz* **2024**, 11, 5631; u)Y. Zhang, T. Li, L. Miao, P. Kaur, S. Men, Q. Wang, X. Gong, Y. Fang, C. Zhai, S. Zhang, L. Zhang, L. Ye, A highly sensitive and ultra-stretchable zwitterionic liquid hydrogel-based sensor as anti-freezing ionic skin, *J Mater Chem A* **2022**, 10, 3970; v)Z. Dai, S. Ding, M. Lei, S. Li, Y. Xu, Y. Zhou, B. Zhou, A superhydrophobic and anti-corrosion strain sensor for robust underwater applications, *J Mater Chem A* **2021**, 9, 15282; w)F. Han, R. Su, L. Teng, R. Xie, Q. Yu, Q. Li, Q. Tian, H. Li, J. Sun, Y. Zhang, M. Li, X. Liu, H. Ye, G. Li, G. Zhang, Z. Liu, Brittle-layer-tuned microcrack propagation for high-performance stretchable strain sensors, *Journal of Materials Chemistry C* **2021**, 9, 7319; x)Y. Ou, T. Zhao, Y. Zhang, G. Zhao, L. Dong, Stretchable solvent-free ionic conductor with self-wrinkling microstructures for ultrasensitive strain sensor, *Mater Horiz* **2022**, 9, 1679; y)B. Feng, X. Jiang, G. Zou, W. Wang, T. Sun, H. Yang, G. Zhao, M. Dong, Y. Xiao, H. Zhu, L. Liu, Nacre-Inspired, Liquid Metal-Based Ultrasensitive Electronic Skin by Spatially Regulated Cracking Strategy, *Advanced Functional Materials* **2021**, 31, 2102359; z)F. Zhuo, J. Zhou, Y. Liu, J. Xie, H. Chen, X. Wang, J. Luo, Y. Fu, A. Elmarakbi, H. Duan, Kirigami-Inspired 3D-Printable MXene Organohydrogels for Soft Electronics, *Advanced Functional Materials* **2023**, 33, 2308487; aa)Y. Shang, C. Huang, Z. Li, X. Du, Bioinspired Ultra‐Stretchable and Highly Sensitive Structural Color Electronic Skins, *Advanced Functional Materials* **2024**, n/a, 2412703; ab)Y. Hu, X. Hao, G. Chen, J. Bian, M. Li, F. Peng, Self-Standing, Photothermal-Actuating, and Motion-Monitoring Janus Films One-Pot Synthesized by Green Carboxymethyl Glucomannan/Liquid Metal Nanoinks, *ACS applied materials & interfaces* **2022**, 14, 23717; ac)Y. Lee, H. Choi, H. Zhang, Y. Wu, D. Lee, W. S. Wong, X. S. Tang, J. Park, H. Yu, K. C. Tam, Sensitive, Stretchable, and Sustainable Conductive Cellulose Nanocrystal Composite for Human Motion Detection, *Acs Sustain Chem Eng* **2021**, 9, 17351; ad)R. Wang, W. Chi, F. Wan, J. Wei, H. Ping, Z. Zou, J. Xie, W. Wang, Z. Fu, Nanocage Ferritin Reinforced Polyacrylamide Hydrogel for Wearable Flexible Strain Sensors, *ACS applied materials & interfaces* **2022**, 14, 21278; ae)Z. Liu, K. Wan, T. Zhu, J. Zhu, J. Xu, C. Zhang, T. Liu, Superelastic, Fatigue-Resistant, and Flame-Retardant Spongy Conductor for Human Motion Detection against a Harsh High-Temperature Condition, *ACS applied materials & interfaces* **2021**, 13, 7580; af)R. Abouzeid, M. Shayan, T. Wu, J. Gwon, T. A. Kärki, Q. Wu, Highly Flexible, Self-Bonding, Self-Healing, and Conductive Soft Pressure Sensors Based on Dicarboxylic Cellulose Nanofiber Hydrogels, *ACS Appl. Polym. Mater.* **2023**, 5, 7009; ag)Z. Gao, M. Bi, Z. Jin, J. Sun, H. Gao, G. Gao, Ultra-adhesive Poly(acrylic acid)-Based Hydrogel as a Flexible Sensor for Capturing Human-Motion Signal, *ACS Appl. Polym. Mater.* **2023**, 5, 1926; ah)A. Lv, X. Lv, Z.-B. Shao, Tailored Tough, Self-Healing, and Antimicrobial Double Cross-Linked Hydrogel Supercapacitor Electrodes with Excellent Capacitive Properties and Stable Conductivity, *Macromolecules* **2024**, 57, 6439; ai)A. Li, J. He, W. Wang, C. Cui, S. Jiang, S. Jiang, W. Qin, C. Cheng, R. Guo, Self-Heating and Hydrophobic Nanofiber Membrane Based on Ti3C2Tx MXene/Ag Nanoparticles/Thermoplastic Polyurethane for Electromagnetic Interference Shielding and Sensing Performance, *Ind. Eng. Chem. Res.* **2022**, 61, 15249; aj)A. Lv, X. Lv, S. Tian, T. Xie, X. Xu, S. Sun, Tough, Self-Healing, and Antimicrobial Hydrogel Sensors Based on Hydrogen-Bonded, Cross-linked Chitosan and MWCNTs, *ACS Appl. Polym. Mater.* **2023**, 5, 6452; ak)J. Yue, Y. Teng, Y. Huang, R. Fan, C. Li, Y. Tao, J. Hu, J. Lu, J. Du, H. Wang, Reduced Graphene Oxide Interlocked Carbonized Loofah for Energy Harvesting and Physiological Signal Monitoring, *ACS Appl. Electron. Mater.* **2024**, 6, 3734; al)Y. Ma, D. Zhang, Z. Wang, H. Zhang, H. Xia, R. Mao, H. Cai, H. Luan, Self-Adhesive, Anti-Freezing MXene-Based Hydrogel Strain Sensor for Motion Monitoring and Handwriting Recognition with Deep Learning, *ACS applied materials & interfaces* **2023**, 15, 29413; am)J. Liang, J. He, Y. Xin, W. Gao, G. Zeng, X. He, MXene Reinforced PAA/PEDOT:PSS/MXene Conductive Hydrogel for Highly Sensitive Strain Sensors, *Macromol. Mater. Eng.* **2023**, 308, 2200519; an)R. P. Verma, P. S. Sahu, M. Rathod, S. S. Mohapatra, J. Lee, B. Saha, Ultra-Sensitive and Highly Stretchable Strain Sensors for Monitoring of Human Physiology, *Macromol. Mater. Eng.* **2022**, 307, 2100666; ao)R. Zeng, C. Qi, S. Lu, J. Xu, C. Zhao, Z. Dong, C. Lei, Hydrophobic association and ionic coordination dual crossed-linked conductive hydrogels with self-adhesive and self-healing virtues for conformal strain sensors, *Journal of Polymer Science* **2022**, 60, 812; ap)S. Srimongkol, P. Wiroonpochit, K. Utra, R. Sethayospongsa, P. Muthitamongkol, B. Methachan, N. Butsri, S. Srisawadi, Carbon-based conductive rubber composite for 3D printed flexible strain sensors, *Polymers for Advanced Technologies* **2023**, 34, 287; aq)D. Li, J. Zhao, X. Guo, J. Jiang, L. Jiang, S. Chen, Y. Zhou, F.-l. Zhou, Core–sheath structured polystyrene-ethylene-butene-styrene/carbon nanotubes/carbonyl iron particlecomposite fiber with high stretchability for strain and magnetic field dual-mode sensing, *Polym Compos* **2024**, 45, 1483; ar)Z. Zeng, S. Yu, C. Guo, D. Lu, Z. Geng, D. Pei, Mxene Reinforced Supramolecular Hydrogels with High Strength, Stretchability, and Reliable Conductivity for Sensitive Strain Sensors, *Macromolecular Rapid Communications* **2022**, 43, 2200103; as)H. Nie, Z. Chen, H. Tang, Y. Ren, W. Liu, Strain sensor based on polyurethane/carbon nanotube elastic conductive spiral yarn with high strain range and sensitivity, *Polym Compos* **2024**, 45, 5522; at)J. Duan, J. Liu, C. Wang, C. Cai, L. Lyu, L. Sun, X. Wu, H. Bi, Stretch-Tolerant Waterproof and Self-cleaning CBNPs/Graphene Strain Sensor for Multifunctional Applications, *Advanced Materials Technologies* **2023**, 8, 2300776; au)D. Fang, Y. Wang, X. Lv, X. Zhang, S. Yi, J. Chen, Y. Ma, W. Xu, X. Yang, H. Jia, Development of a Nano-toughened multifunctional composite hydrogel based on chitosan and its applications in catalytic and flexible sensors, *International Journal of Biological Macromolecules* **2024**, 139016; av)S. Y. Lee, H. Lim, H. J. Choi, J. Ahn, Y. K. Choi, D. Y. Oh, D. Chae, H. Lee, S. J. Oh, Designing comfortable-to-use wearable strain sensors with thermal management through radiative cooling function, *Chemical Engineering Journal* **2024**, 498, 155691; aw)Y. Lou, J. Wang, Y. Peng, X. Wang, J. Zhang, L. Chen, W. Gao, Z. Gao, X. Li, W. Chen, M. Zhao, Double network hydrogel confined MXene/Liquid metal by dynamic hydrogen bond for high-performance wearable sensors, *Chemical Engineering Journal* **2024**, 500, 156884; ax)K. Fan, K. Li, L. Han, Z. Yang, J. Yang, J. Zhang, J. Cheng, Multifunctional double-network Ti3C2Tx MXene composite hydrogels for strain sensors with effective electromagnetic interference and UV shielding properties, *Polymer* **2023**, 273, 125865; ay)S. Tu, Y. Ma, L. Shi, H. Li, M. Chen, L. Wu, Aligned silver Nanowires/Polymer composite films for ultrasensitive and highly stretchable strain sensors, *Chemical Engineering Journal* **2023**, 473, 145075; az)Y. Yang, L. Kong, J. Lu, B. Lin, L. Fu, C. Xu, A highly conductive MXene-based rubber composite with relatively stable conductivity under small deformation and high sensing sensitivity at large strain, *Composites Part A: Applied Science and Manufacturing* **2023**, 170, 107545; ba)J. Wu, D. Xu, Z. Feng, L. Zhu, C. Dong, J. Qiu, Stretchable and highly sensitive polyelectrolyte microgels-enhanced polyacrylamide hydrogel composite for strain sensing, *Composites Communications* **2022**, 35, 101332.

[9] a)D. H. Lee, T. Miyashita, Y. Xuan, K. Takei, Ultrasensitive and Stretchable Strain Sensors Based on Laser-Induced Graphene With ZnO Nanoparticles, *ACS Nano* **2024**, 18, 32255; b)P. Li, H. Wang, Z. Ju, Z. Jin, J. Ma, L. Yang, X. Zhao, H. Xu, Y. Liu, Ti3C2Tx MXene- and Sulfuric Acid-Treated Double-Network Hydrogel with Ultralow Conductive Filler Content for Stretchable Electromagnetic Interference Shielding, *ACS Nano* **2024**, 18, 2906; c)G. Su, N. Wang, Y. Liu, R. Zhang, Z. Li, Y. Deng, B. Z. Tang, From Fluorescence-Transfer-Lightening-Printing-Assisted Conductive Adhesive Nanocomposite Hydrogels toward Wearable Interactive Optical Information-Electronic Strain Sensors, *Advanced Materials* **2024**, 36, 2400085; d)Y. Liu, Z. Xu, X. Ji, X. Xu, F. Chen, X. Pan, Z. Fu, Y. Chen, Z. Zhang, H. Liu, B. Cheng, J. Liang, Ag–thiolate interactions to enable an ultrasensitive and stretchable MXene strain sensor with high temporospatial resolution, *Nature Communications* **2024**, 15, 5354; e)T. Li, H. Qi, Y. Zhao, P. Kumar, C. Zhao, Z. Li, X. Dong, X. Guo, M. Zhao, X. Li, X. Wang, R. O. Ritchie, W. Zhai, Robust and sensitive conductive nanocomposite hydrogel with bridge cross-linking–dominated hierarchical structural design, *Science Advances* **2024**, 10, eadk6643; f)E. Khakpour, S. Sadeghzadeh, Wearable gold decorated direct laser writing graphene for ultra-minor strains, *Physical Chemistry Chemical Physics* **2024**, 26, 26871; g)H. Wu, H. Qi, X. Wang, Y. Qiu, K. Shi, H. Zhang, Z. Zhang, W. Zhang, Y. Tian, Stretchable, sensitive, flexible strain sensor incorporated with patterned liquid metal on hydrogel for human motion monitoring and human–machine interaction, *Journal of Materials Chemistry C* **2022**, 10, 8206; h)S. Han, Y. Hu, J. Wei, S. Li, P. Yang, H. Mi, C. Liu, C. Shen, A Semi-Interpenetrating Poly(Ionic Liquid) Network-Driven Low Hysteresis and Transparent Hydrogel as a Self-Powered Multifunctional Sensor, *Advanced Functional Materials* **2024**, 34, 2401607; i)Q. Ling, W. Liu, J. Liu, L. Zhao, Z. Ren, H. Gu, Highly Sensitive and Robust Polysaccharide-Based Composite Hydrogel Sensor Integrated with Underwater Repeatable Self-Adhesion and Rapid Self-Healing for Human Motion Detection, *ACS applied materials & interfaces* **2022**, 14, 24741; j)J. Ji, C. Zhang, S. Yang, Y. Liu, J. Wang, Z. Shi, High Sensitivity and a Wide Sensing Range Flexible Strain Sensor Based on the V-Groove/Wrinkles Hierarchical Array, *ACS applied materials & interfaces* **2022**, 14, 24059; k)L. Kang, J. Ma, C. Wang, K. Li, H. Wu, M. Zhu, Highly Sensitive and Wide Detection Range Thermoplastic Polyurethane/Graphene Nanoplatelets Multifunctional Strain Sensor with a Porous and Crimped Network Structure, *ACS applied materials & interfaces* **2024**, 16, 2814; l)H. Dong, J. Sun, X. Liu, X. Jiang, S. Lu, Highly Sensitive and Stretchable MXene/CNTs/TPU Composite Strain Sensor with Bilayer Conductive Structure for Human Motion Detection, *ACS applied materials & interfaces* **2022**, 14, 15504; m)S. Han, H. Tan, J. Wei, H. Yuan, S. Li, P. Yang, H. Mi, C. Liu, C. Shen, Surface Modification of Super Arborized Silica for Flexible and Wearable Ultrafast-Response Strain Sensors with Low Hysteresis, *Advanced Science* **2023**, 10, 2301713; n)J. Liu, D. Wu, C. Liu, Q. Wang, H. Wang, Full-Range On-Body Strain Sensor of Laser-Induced Graphene Embedded in Thermoplastic Elastomer via Hot Pressing Transfer for Monitoring of the Physiological Signals, *Advanced Materials Technologies* **2024**, 9, 2301658; o)I. Ijaz, A. Bukhari, E. Gilani, A. Nazir, H. Zain, A. Shaheen, M. R. Shaik, M. E. Assal, M. Khan, MXene, protein, and KCl-assisted ionic conductive hydrogels with excellent anti-freezing capabilities, self-adhesive, ultra-stretchability, and remarkable mechanical properties for a high-performance wearable flexible sensor, *Rsc Adv* **2024**, 14, 21786.

[10] a)T. Li, H. Qi, X. Dong, G. Li, W. Zhai, Highly Robust Conductive Organo-Hydrogels with Powerful Sensing Capabilities Under Large Mechanical Stress, *Advanced Materials* **2024**, 36, 2304145; b)H. Yu, Q. Zhuang, J. Lin, Z. Chen, Z. Chen, Z. Wang, G. Zhou, S. Zhang, Y. Lai, D. Wu, One-step fabrication of high-performance graphene composites from graphite solution for bio-scaffolds and flexible strain sensors, *Nanotechnology* **2023**, 34, 315301; c)P. Karipoth, A. Pullanchiyodan, A. Christou, R. Dahiya, Graphite-Based Bioinspired Piezoresistive Soft Strain Sensors with Performance Optimized for Low Strain Values, *ACS applied materials & interfaces* **2021**, 13, 61610; d)X. Shi, H. Wang, X. Xie, Q. Xue, J. Zhang, S. Kang, C. Wang, J. Liang, Y. Chen, Bioinspired Ultrasensitive and Stretchable MXene-Based Strain Sensor via Nacre-Mimetic Microscale “Brick-and-Mortar” Architecture, *ACS Nano* **2019**, 13, 649; e)J. Cao, Z. Zhang, L. Wang, T. Lin, H. Li, Q. Zhao, H. Wang, X. Liu, H. Yang, B. Lu, An adhesive, highly stretchable and low-hysteresis alginate-based conductive hydrogel strain sensing system for motion capture, *International Journal of Biological Macromolecules* **2024**, 281, 136581.

[11] a)A. a. Al-Halhouli, A. Albagdady, A. Rabadi, M. Hamdan, J. Abu-Khalaf, M. Abu-Abeeleh, Screen-printed wearable sensors for continuous respiratory rate monitoring: fabrication, clinical evaluation, and point-of-care potential, *Mater. Adv.* **2024**, 5, 9586; b)Y. Hao, Q. Yan, H. Liu, X. He, P. Zhang, X. Qin, R. Wang, J. Sun, L. Wang, Y. Cheng, A Stretchable, Breathable, And Self-Adhesive Electronic Skin with Multimodal Sensing Capabilities for Human-Centered Healthcare, *Advanced Functional Materials* **2023**, 33, 2303881; c)S. J. Paul, I. Elizabeth, B. K. Gupta, Ultrasensitive Wearable Strain Sensors based on a VACNT/PDMS Thin Film for a Wide Range of Human Motion Monitoring, *ACS applied materials & interfaces* **2021**, 13, 8871; d)J. Huang, Z. Li, T. Kang, W. Wei, F. Liu, X. Xu, Z. Liu, Fabrication of styrene–butadienestyrene (SBS) matrix-based flexible strain sensors with brittle cellulose nanocrystal (CNC)/carbon black (CB) segregated networks, *Composite Structures* **2023**, 320, 117231; e)J. Ma, Y. Qing, H. Song, X. Cheng, Z. Li, C. Long, C. Liu, Synergistically coupled double conductive coating-based electronic textiles with superhydrophobic and high-performance strain sensing properties for underwater human motion sensing applications, *Chemical Engineering Journal* **2023**, 471, 144284.

[12] F. K. Abd Hamid, M. N. Hasan, G. E. Murty, M. I. Ahmad Asri, T. Saleh, M. S. Mohamed Ali, Resistive strain sensors based on carbon black and multi-wall carbon nanotube composites, *Sensors and Actuators A: Physical* **2024**, 366, 114960.

[13] Z. Wang, H. Zhu, H. Li, Z. Wang, M. Sun, B. Yang, Y. Wang, L. Wang, L. Xu, High-Strength Magnetic Hydrogels with Photoweldability Made by Stepwise Assembly of Magnetic-Nanoparticle-Integrated Aramid Nanofiber Composites, *ACS Nano* **2023**, 17, 9622.

[14] T. Chang, S. Akin, S. Cho, J. Lee, S. A. Lee, T. Park, S. Hong, T. Yu, Y. Ji, J. Yi, S. L. Gong, D. R. Kim, Y. L. Kim, M. B.-G. Jun, C. H. Lee, In Situ Spray Polymerization of Conductive Polymers for Personalized E-textiles, *ACS Nano* **2023**, 17, 22733.

[15] W. Dou, M. Malhi, T. Cui, M. Wang, T. Wang, G. Shan, J. Law, Z. Gong, J. Plakhotnik, T. Filleter, R. Li, C. A. Simmons, J. T. Maynes, Y. Sun, A Carbon-Based Biosensing Platform for Simultaneously Measuring the Contraction and Electrophysiology of iPSC-Cardiomyocyte Monolayers, *ACS Nano* **2022**, 16, 11278.

[16] P. Nazari, R. Bäuerle, J. Zimmermann, C. Melzer, C. Schwab, A. Smith, W. Kowalsky, J. Aghassi-Hagmann, G. Hernandez-Sosa, U. Lemmer, Piezoresistive Free-standing Microfiber Strain Sensor for High-resolution Battery Thickness Monitoring, *Advanced Materials* **2023**, 35, 2212189.

[17] Y. Zhou, C. Yu, X. Zhang, Y. Zheng, B. Wang, Y. Bao, G. Shan, H. Wang, P. Pan, Ultrasensitive Ionic Conductors with Tunable Resistance Switching Temperature Enabled by Phase Transformation of Polymer Cocrystals, *Advanced Materials* **2024**, 36, 2309568.

[18] J. M. Morales Ferrer, R. E. Sánchez Cruz, S. Caplan, W. M. van Rees, J. W. Boley, Multiscale Heterogeneous Polymer Composites for High Stiffness 4D Printed Electrically Controllable Multifunctional Structures, *Advanced Materials* **2024**, 36, 2307858.

[19] J. Pu, Q. Cao, Y. Gao, Q. Wang, Z. Geng, L. Cao, F. Bu, N. Yang, C. Guan, Liquid Metal-Based Stable and Stretchable Zn-Ion Battery for Electronic Textiles, *Advanced Materials* **2024**, 36, 2305812.

[20] S. Wu, Z. Liu, C. Gong, W. Li, S. Xu, R. Wen, W. Feng, Z. Qiu, Y. Yan, Spider-silk-inspired strong and tough hydrogel fibers with anti-freezing and water retention properties, *Nature Communications* **2024**, 15, 4441.

[21] S. Hao, Q. Fu, L. Meng, F. Xu, J. Yang, A biomimetic laminated strategy enabled strain-interference free and durable flexible thermistor electronics, *Nature Communications* **2022**, 13, 6472.

[22] F. Sun, L. Liu, T. Liu, X. Wang, Q. Qi, Z. Hang, K. Chen, J. Xu, J. Fu, Vascular smooth muscle-inspired architecture enables soft yet tough self-healing materials for durable capacitive strain-sensor, *Nature Communications* **2023**, 14, 130.

[23] Y. M. Kim, J. H. Kwon, S. Kim, U. H. Choi, H. C. Moon, Ion-cluster-mediated ultrafast self-healable ionoconductors for reconfigurable electronics, *Nature Communications* **2022**, 13, 3769.

[24] X. Zhang, M. Cui, S. Wang, F. Han, P. Xu, L. Teng, H. Zhao, P. Wang, G. Yue, Y. Zhao, G. Liu, K. Li, J. Zhang, X. Liang, Y. Zhang, Z. Liu, C. Zhong, W. Liu, Extensible and self-recoverable proteinaceous materials derived from scallop byssal thread, *Nature Communications* **2022**, 13, 2731.

[25] P. Xu, S. Wang, A. Lin, H.-K. Min, Z. Zhou, W. Dou, Y. Sun, X. Huang, H. Tran, X. Liu, Conductive and elastic bottlebrush elastomers for ultrasoft electronics, *Nature Communications* **2023**, 14, 623.

[26] S. Li, H. Wang, W. Ma, L. Qiu, K. Xia, Y. Zhang, H. Lu, M. Zhu, X. Liang, X.-E. Wu, H. Liang, Y. Zhang, Monitoring blood pressure and cardiac function without positioning via a deep learning–assisted strain sensor array, *Science Advances* **2023**, 9, eadh0615.

[27] L. Dong, M. Ren, Y. Wang, G. Wang, S. Zhang, X. Wei, J. He, B. Cui, Y. Zhao, P. Xu, X. Wang, J. Di, Q. Li, Artificial neuromuscular fibers by multilayered coaxial integration with dynamic adaption, *Science Advances* **2022**, 8, eabq7703.

[28] S. Li, X. Chen, X. Li, H. Tian, C. Wang, B. Nie, J. He, J. Shao, Bioinspired robot skin with mechanically gated electron channels for sliding tactile perception, *Science Advances* **2022**, 8, eade0720.

[29] Z. Li, B. Li, B. Chen, J. Zhang, Y. Li, 3D printed graphene/polyurethane wearable pressure sensor for motion fitness monitoring, *Nanotechnology* **2021**, 32, 395503.

[30] V. Selamneni, T. Akshaya, V. Adepu, P. Sahatiya, Laser-assisted micropyramid patterned PDMS encapsulation of 1D tellurium nanowires on cellulose paper for highly sensitive strain sensor and its photodetection studies, *Nanotechnology* **2021**, 32, 455201.

[31] D. Lei, H. Zhang, N. Liu, Q. Zhang, T. Su, L. Wang, Z. Ren, Z. Zhang, J. Su, Y. Gao, Tensible and flexible high-sensitive spandex fiber strain sensor enhanced by carbon nanotubes/Ag nanoparticles, *Nanotechnology* **2021**, 32, 505509.

[32] X. F. Sánchez-Romate, É. Gómez, M. Sánchez, A. Ureña, Carbon nanoparticle reinforced adhesive films as surface sensors for strain detection, *Nanotechnology* **2023**, 34, 26LT01.

[33] Y. K. Choi, T. Park, D. H. D. Lee, J. Ahn, Y. H. Kim, S. Jeon, M. J. Han, S. J. Oh, Wearable anti-temperature interference strain sensor with metal nanoparticle thin film and hybrid ligand exchange, *Nanoscale* **2022**, 14, 8628.

[34] C. M. Palicpic, R. Khadka, J.-H. Yim, An effectively enhanced vapor phase hybridized conductive polymer based on graphene oxide and glycerol influence for strain sensor applications, *New J. Chem.* **2022**, 46, 22162.

[35] Y. Zhu, J. Liu, M. Ou, L. Dai, W. Zhang, J. Wang, J. Sun, C. Qin, L. Dai, A spring-like fiber based strain sensor with a fast response and high sensitivity for precise detection of complicated human activities, *Journal of Materials Chemistry C* **2024**, 12, 18077.

[36] J. S. Cho, W. Jang, K. H. Park, D. H. Wang, Thermally stable metallic glass strain sensors with extended sensing range and sensitivity, *Journal of Materials Chemistry C* **2024**, 12, 7532.

[37] Z. Liu, Y. Zheng, L. Jin, K. Chen, H. Zhai, Q. Huang, Z. Chen, Y. Yi, M. Umar, L. Xu, G. Li, Q. Song, P. Yue, Y. Li, Z. Zheng, Highly Breathable and Stretchable Strain Sensors with Insensitive Response to Pressure and Bending, *Advanced Functional Materials* **2021**, 31, 2007622.

[38] Y. Liang, Q. Song, Y. Chen, C. Hu, S. Zhang, Stretch-Induced Robust Intrinsic Antibacterial Thermoplastic Gelatin Organohydrogel for a Thermoenhanced Supercapacitor and Mono-gauge-factor Sensor, *ACS applied materials & interfaces* **2023**, 15, 20278.

[39] Y. Li, H. Peng, Y. Peng, J. Zhou, J. Zhang, Thermoplastic and Electrically Conductive Fibers for Highly Stretchable and Sensitive Strain Sensors, *ACS Appl. Polym. Mater.* **2022**, 4, 8795.

[40] S. Chen, R. Wu, P. Li, Q. Li, Y. Gao, B. Qian, F. Xuan, Acid-Interface Engineering of Carbon Nanotube/Elastomers with Enhanced Sensitivity for Stretchable Strain Sensors, *ACS applied materials & interfaces* **2018**, 10, 37760.

[41] M. Solazzo, L. Hartzell, C. O’Farrell, M. G. Monaghan, Beyond Chemistry: Tailoring Stiffness and Microarchitecture to Engineer Highly Sensitive Biphasic Elastomeric Piezoresistive Sensors, *ACS applied materials & interfaces* **2022**, 14, 19265.

[42] A. M. Barja, Y. K. Ryu, S. Tarancón, E. Tejado, A. Hamada, A. Velasco, J. Martinez, Laser-Induced Graphene Strain Sensors for Body Movement Monitoring, *ACS Omega* **2024**, 9, 38359.

[43] X. Cheng, J. Cai, J. Xu, D. Gong, High-Performance Strain Sensors Based on Au/Graphene Composite Films with Hierarchical Cracks for Wide Linear-Range Motion Monitoring, *ACS applied materials & interfaces* **2022**, 14, 39230.

[44] W. Zhu, M. Wang, Z. Zhang, J. Sun, J. Zhan, M. Guan, Z. Xu, S. Wang, X. Li, L. Jiang, Controllable Photoreduction of Graphene Oxide/Gold Composite Using a Shaped Femtosecond Laser for Multifunctional Sensors, *ACS applied materials & interfaces* **2023**, 15, 52883.

[45] J. Cui, J. Chen, Z. Ni, W. Dong, M. Chen, D. Shi, High-Sensitivity Flexible Sensor Based on Biomimetic Strain-Stiffening Hydrogel, *ACS applied materials & interfaces* **2022**, 14, 47148.

[46] J. Fernández Maestu, A. García Díez, C. R. Tubio, A. Gómez, J. Berasategui, P. Costa, M. M. Bou-Ali, J. G. Etxebarria, S. Lanceros-Méndez, Ternary Multifunctional Composites with Magnetorheological Actuation and Piezoresistive Sensing Response, *ACS Appl. Electron. Mater.* **2023**, 5, 4296.

[47] G. Huang, P. Wang, Y. Cai, K. Jiang, H. Li, Tough, self-healing double network hydrogels crosslinked via multiple dynamic non-covalent bonds for strain sensor, *Journal of Polymer Science* **2023**, 61, 1675.

[48] W. Wang, X. Deng, C. Luo, Anisotropic strain sensors to realize skin-comparable performances, *J. Appl. Polym. Sci.* **2024**, 141, e54829.

[49] W. Xu, M. A. A. Newton, Z. Chen, B. Xin, Design, characterization, and performance evaluation of novel PVA/CS/CNF/MOP TN ionic conductive hydrogels for flexible sensors, *Journal of Polymer Science* **2024**, 62, 2744.

[50] K. Orikasa, L. Benedetti, C. Park, S.-H. Chu, A. Jimenez, T. Dolmetsch, T. Thomas, A. Agarwal, Smart Foams: Boron Nitride-Graphene Nanoplatelet Foams for Tunable Radiation Shielding and Strain Sensing, *Advanced Materials Technologies* **2024**, 9, 2400106.

[51] V. Adepu, K. Kamath, S. Siddhartha, V. Mattela, P. Sahatiya, MXene/TMD Nanohybrid for the Development of Smart Electronic Textiles Based on Physical Electromechanical Sensors, *Advanced Materials Interfaces* **2022**, 9, 2101687.

[52] S. Ahmed, M. Bodaghi, S. Nauman, Z. Muhammad Khan, Additive Manufacturing of Flexible Strain Sensors Based on Smart Composites for Structural Health Monitoring with High Accuracy and Fidelity, *Advanced Engineering Materials* **2023**, 25, 2300763.

[53] J. J. Andrew, M. A. Uddin, S. Kumar, A. Schiffer, Mechanical and piezoresistive performance of additively manufactured carbon fiber/PA12 hybrid honeycombs, *Thin-Walled Structures* **2024**, 201, 111950.

[54] H. N. Fath Dehghan, A. Abdolmaleki, M. Pourahmadi, S. Hozori, E. Gaeini, S. Y. Mousavi, A.-R. Arvaneh, M. Sadat-Shojai, Ultra-strength and anti-freezing zwitterionic hydrogels with high ion conductivity: Effect of the hydrophobic monomer in hydrogels mechanical properties, *Polym Test* **2024**, 140, 108607.

[55] K. A. Dubey, R. K. Mondal, Y. K. Bhardwaj, Graphene assisted enhancement in the cyclic electromechanical properties of polyolefin based multiphasic conducting nano carbon black nanocomposites, *Radiation Physics and Chemistry* **2024**, 214, 111308.

[56] Y. Z. N. Htwe, M. Mariatti, Performance of water-based printed hybrid graphene/silver nanoparticle conductive inks for flexible strain sensor applications, *Synthetic Metals* **2023**, 300, 117495.

[57] X. Liu, L. Wei, X. Wang, S. He, Y. Yan, Q. Li, H. Yang, C. Hu, Y. Ling, Flexible strain sensors based on gold nanowire dominoes for human motion detection, *Materials Today Communications* **2023**, 35, 105703.

[58] M. Asad Ullah Khalid, S. Hwan Chang, Characterization of highly linear stretchable sensor made of Gr-PEDOT:PSS/MnO2 nanowires/Ecoflex composite, *Composite Structures* **2023**, 311, 116824.

[59] X. Qi, W. Wang, H. Dai, Y. Zhu, Y. Dong, S.-Y. Fu, Q. Ni, Y. Fu, Multifunctional two-way shape memory RGO/ethylene-vinyl acetate composite yarns for electro-driven actuators and high sensitivity strain sensors, *Composites Part A: Applied Science and Manufacturing* **2023**, 169, 107521.
